# Cultivation of filamentous fungi in airlift bioreactors: advantages and disadvantages

**DOI:** 10.1007/s00253-025-13422-4

**Published:** 2025-02-10

**Authors:** Federico Cerrone, Kevin E. O’Connor

**Affiliations:** 1 School of Biotechnology, Dublin City University, Glasnevin Campus Dublin, Dublin, Ireland; 2 BiOrbic Bioeconomy Research Centre, O’Brien Centre for Science (Science East) University College Dublin, Belfield Campus Dublin, Dublin, Ireland; 3 School of Biomolecular and Biomedical Sciences, University College Dublin, Belfield Campus Dublin, Dublin, Ireland; 4 Bioplastech Ltd NovaUCD, Belfield Innovation Park, University College Dublin, Dublin, Ireland

**Keywords:** Airlift fermentation, Filamentous fungi, Volumetric productivity, Fluid dynamics

## Abstract

**Abstract:**

Filamentous fungi or mycelia are a valuable bioresource to produce several biomolecules and enzymes, especially because of their biodegradation potential and for their key role of enablers of a circular bioeconomy. Filamentous fungi can be grown in submerged cultivation to maximise the volumetric productivity of the bioprocess, instead of using the more established and time-consuming solid-state cultivation. Multicellular mycelia are sensitive to shear stresses induced by mechanical agitation, and this aspect greatly affects their morphology in submerged cultivation (pelletisation) and the connected volumetric productivity. An efficient compromise is the growth of filamentous fungi in airlift bioreactors (ALR) where the volumetric oxygen transfer (K_L_a) is optimal, but the shear stress is reduced. In this review, we critically analysed the advantages and disadvantages of ALR-based cultivation of filamentous fungi, comparing these bioreactors also with stirred tank reactors and bubble column reactors; we focused on scientific literature that highlights findings for the cultivation of filamentous fungi for both the production of enzymes and the production of myco-biomass in ALR; we included studies for the control of the pelletisation of the fungal biomass in batch and semi-continuous cultivation, highlighting the interlinked hydrodynamics; finally, we included studies regarding the modifications of ALR in order to enhance filamentous fungi production.

**Key points:**

*• ALR are efficient for batch and prolonged continuous cultivation of filamentous fungi.*

*• ALR show both optimal gas hold-up and K*_*L*_ *a with an airflow that has high superficial velocity and critical bubble diameter (1–6 mm).*

*• Suspended mycelia aggregates (pellet) maintain a fluidised motion in ALR if their size/density can be controlled.*

## Introduction

### Filamentous fungi morphology and growth phases

Mycelia is another term to describe the filamentous morphology of fungi that are composed of hyphal cells. Mycelia can be constituted by simple septate hyphae (in *Ascomycota*), where the hyphal cells are monokaryotic for most of the life cycle of their vegetative growth. *Basidiomycota* have instead specialised dolipore septa and clamp connections between adjacent hyphal cells (Kües 2000). Vegetative growth of monokaryotic hyphal cells of *Basidiomycota* will allow the mating of other hyphae (sexual reproduction) in either homothallic (self-haploid mating) or heterothallic (not-self-haploid mating) (Kües 2000). Most of the filamentous fungi of edible interest (subphylum: *Agaricomycotina*, class *Agaricomycetes*) have heterothallic hyphae with a strictly regulated sexual reproduction (Coelho et al. 2017). The sexual reproduction is followed by the plasmogamy (fusion of the cytoplasm of the adjacent hyphal cells) and therefore the resulting dikaryotic morphology; this event is separated in time from the subsequent karyogamy, because the *Basidiomycota* evolved a peculiar mechanism of differentiation of an adequate dikaryotic aggregation of hyphal cell, known as basidiocarp. This morphological structure can have multiple shapes (the well-known mushroom cap shape is one of them), and it is the morphology that is conducive to allow the basidia formation; these basidia are the locations where the basidiospores (at this stage with a diploid nucleus) are formed. When the basidiospores undergo a meiosis process and a meiosis crossover (which is not randomly occurring across the chromosomal organisation of the genome) (Gong et al. 2021), the subsequent dispersion of haploid basidiospores generates new haploid hyphae that start a new vegetative growth. Hyphal vegetative growth is therefore an environmental adaptation by which filamentous fungi make the most of their ability to spread and maximise the uptake of nutrients from the surrounding environment. Due to their lifestyle, fungi are strictly associated with plants by a dynamic interaction; filamentous mushroom-forming fungi in the wild can be described as saprotrophs or biotrophs, either capable of degrading complex and lignocellulosic polymers and/or opportunists that obtain carbon compounds from their mutualistic host, providing a key-ecosystemic service of inorganic nutrient recirculation (Kohler et al. 2015). Many of them maintain an ectomycorrhizal adaptation and variedly expressed plant cell wall degrading enzymes (PCWDE) (Kohler et al. 2015). The environmental triggers that promote the formation of fruiting bodies (basidiocarps) are mainly blue and UV light sensing, decreasing overnight temperature, high localised relative humidity, reduced carbon dioxide concentration and nutrient starvation (Sakamoto 2018); many of these factors trigger species-specific responses whose interplay are still the object of research (Sakamoto 2018, Chadwick and Lin 2024). Phototropism inducing the stipe elongation is maybe the most well-known and remarkable of the environmental triggers (Liu et al. 2021). The other one is gravitropism (Kern 1999).

Traditionally, the mushroom-forming filamentous fungi have been grown in solid-state fermentation, usually on compost or sawdust substrate; the disadvantage of this approach is the length of the process (duration of weeks/months) and the cumbersome control of the environmental triggers to facilitate fruiting bodies formation. Conversely, submerged cultivation of vegetative multicellular hyphae (mycelia) has the advantage of maximising mass transfer (oxygen and nutrients) and therefore reducing cultivation time, potentially increasing volumetric productivity of the bioprocess (Cerrone et al. 2024); many researchers opted to utilise airlift bioreactors for the cultivation of filamentous fungi, due to these organisms suffering shear stress by mechanical agitation (Nitayavardhana et al. 2013; Souza Filho et al. 2017; Cerrone et al. 2024). Filamentous fungi grow in a pelletised morphology when cultivated in submerged fermentation. Each individual pellet can be approximated to a spherical particle of different volume and size for modelling purposes. The filamentous fungi grow according to an exponential growth curve until the density of the pellet causes limitation of oxygen or nutrient transfer to the fungal biomass; this exponential growth has only been associated with the actively dividing tip of the hyphal ends of the spherical pellets. This aspect is particularly important for the volumetric oxygen mass transfer to the pellet of gas to solid molecular diffusion. In its entirety, the pellets grow according to a linearly increasing cubic root growth model (Pirt 1966; Cui et al. 2000), following the Eq. (1):

1 $${M}_{t}^{1/3}=kt+ {M}_{0}^{1/3}$$

where *M*_0_ is the value of the concentration of biomass at time 0 and *M*_*t*_ is the concentration of the biomass at time *t*. *k* is a constant of proportionality (g^1/3^/L^1/3^/day). On this key aspect, Tang and Zhong (2003) proposed an Eq. (2) that correlates the critical pellet density with the dissolved oxygen transfer (DOT) and with the specific oxygen uptake rate (SOUR) by the fungal pellet.

2 $${d}_{\mathrm{crit}}=\sqrt{\frac{24\times {C}_{{\mathrm{O}}_{2}}\times {D}_{\mathrm{eff}}}{{R}_{{\mathrm{O}}_{2}}}}$$

where *d*_crit_ is the critical pellet diameter at which the internal oxygen limitation will occur, *C*_O2_ is the DOT in the medium, *D*_eff_ is the effective diffusion coefficient of oxygen in mycelial pellets, and *R*_O2_ is the oxygen consumption rate per pellet volume. *D*_eff_ is given by the product of the molecular diffusion coefficient with the pellet porosity (1.9 × 10^−9^ m^2^/s). Therefore, the gradient of oxygen concentration that is promoting oxygen diffusion through the pellet is counteracted by the increasing thickness of the pellet, according to the Fick’s first law of diffusion (3).

3 $$J=-D\frac{d\varphi }{dx}$$

where *J* is the mass transfer rate, *D* is the (oxygen) diffusion coefficient, *dφ* is the oxygen concentration, and *dx* is the pellet thickness. Besides the above-mentioned cubic root growth model (that linearly correlates the total fungal biomass to the initial biomass concentration, using a macroscopic approach), other complex and accurate kinetic models are also worth of notice; these models are usually obtained by a combination of experimental data and computational simulations; improvements in the complexity of the software behind the modelling evolved through the years along the improvements in information technology; this aspect allowed to obtain a clearer picture of the dynamics interplaying behind the morphology of filamentous fungi when cultivated in submerged fermentation (especially by including individual morphological changes in actively growing cells). A particularly successful model to describe the fungal growth is a structured model that includes the three main types of cells that belong to the vegetative mycelia (apical, subapical, and hyphal cells) (Nielsen 1993); the rationale behind this model implies that the actively growing cells are only the apical and subapical cells and that when an apical cells emerges from a subapical cell, it is considered a branching point of the mycelia. The growth of individual apical and subapical cells is described by a Monod model linearly correlated to the individual substrate uptake by the cell (4).

4 $$\begin{array}{c}{\upmu }_{\mathrm{a}}={\mathrm{k}}_{\mathrm{a}}\mathrm{s}/\left(\mathrm{s}+{\mathrm{K}}_{\mathrm{s}}\right)\\ {\upmu }_{\mathrm{s}}={\mathrm{k}}_{\mathrm{s}}\mathrm{s}/\left(\mathrm{s}+{\mathrm{K}}_{\mathrm{s}}\right)\\ {\upmu }_{\mathrm{h}}=0\end{array}$$

The mass of a hyphal growth unit (*m*_hgu_) can be defined as the ratio of the total mass of the mycelia divided by the number of actively growing tips. The mechanism of growth in the apical cells is a carefully regulated sequence of vacuoles and peroxisomes translocation (Steinberg 2016), facilitated by kinesin/dynein and microtubuli, revolving around the role of the polarising *Spitzenkörper* (Fisher and Roberson 2016; Riquelme et al. 2018); this protoplasmic movement, in a constantly chitin-reinforced cell wall, resembles the amoeboid chemotaxis and could justify the phylogenetic proximity between fungi and animalia (Brent Heath and Steinberg 1999). This microscopic-focused modelling approach (Nielsen 1992) is very successful in equating the *m*_hgu_ as the ratio of the specific growth rate (*µ*) with the specific branching frequency (*ɸ*); it is found that *m*_hgu_ is constant for most of the filamentous fungi. Knowing the diameter of the hyphae (*d*), the length of the hyphal growth unit (*l*_hgu_) can be calculated by the following Eq. (5) (where *w* is the water content of the hypha, *ρ* is the density of the mycelium, and *V*_hgu_ is the volume of the hyphal growth unit):

5 $$\begin{array}{cc}{\mathrm{m}}_{\mathrm{hgu}}=\rho \left(1-w\right)\frac{\pi }{4}{d}^{2}{l}_{hgu}& \mathrm{Where},{\mathrm{V}}_{\mathrm{hgu}}=\frac{\pi }{4}{d}^{2}{l}_{hgu}\end{array}$$

Moreover, the rate of tip extension (*r*_tip_ = the hyphal mass formed per tip per unit of time) is given as the product of *m*_hgu_ and the total specific growth rate (*µ*). Even if the submerged cultivation of filamentous fungi allows a potentially unlimited nutrient diffusion (Schmideder et al. 2019) to the individual apical cells, the biosynthesis of the fungal biomass is directly correlated to the oxygen availability (as mentioned above); the increasing density of the pellets creates a clear oxygen gradient among concentrical layers of fungal biomass that make the single pellets. A macroscopic cubic root model of biomass production is a simplified approach integrating all the different models that describe the dynamics interplaying in the submerged cultivation of filamentous fungi, similar to Lejeune and Baron (1998). The trade-off between losing the basidiocarp (fruiting body) formation when the fungal biomass is cultivated in submerged media is compensated by the increase in volumetric productivity of biomass production due to higher nutrient/oxygen transfer from the media to the fungal pellets; in fact, the filamentous fungi can be grown to a higher density, without the need for gravitropism. Evaluating different strategies for submerged cultivation of filamentous fungi (especially *Basidiomycota*) in airlift bioreactors is the objective of this mini-review.

## Different types of airlift bioreactor designs

**Fig. 1:**
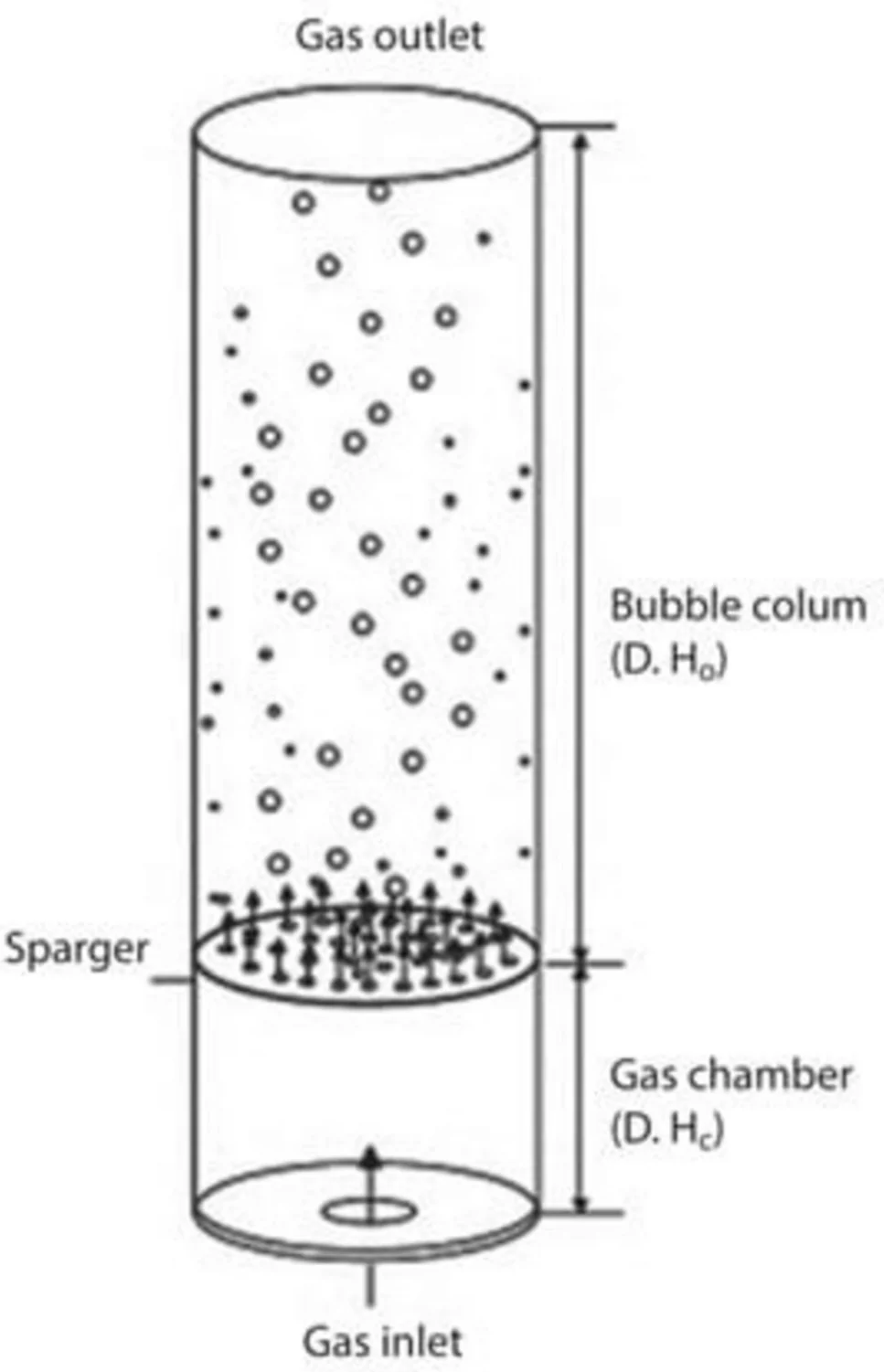
Bubble column bioreactor. Picture adapted from Singhal et al. (2018)

**Fig. 2:**
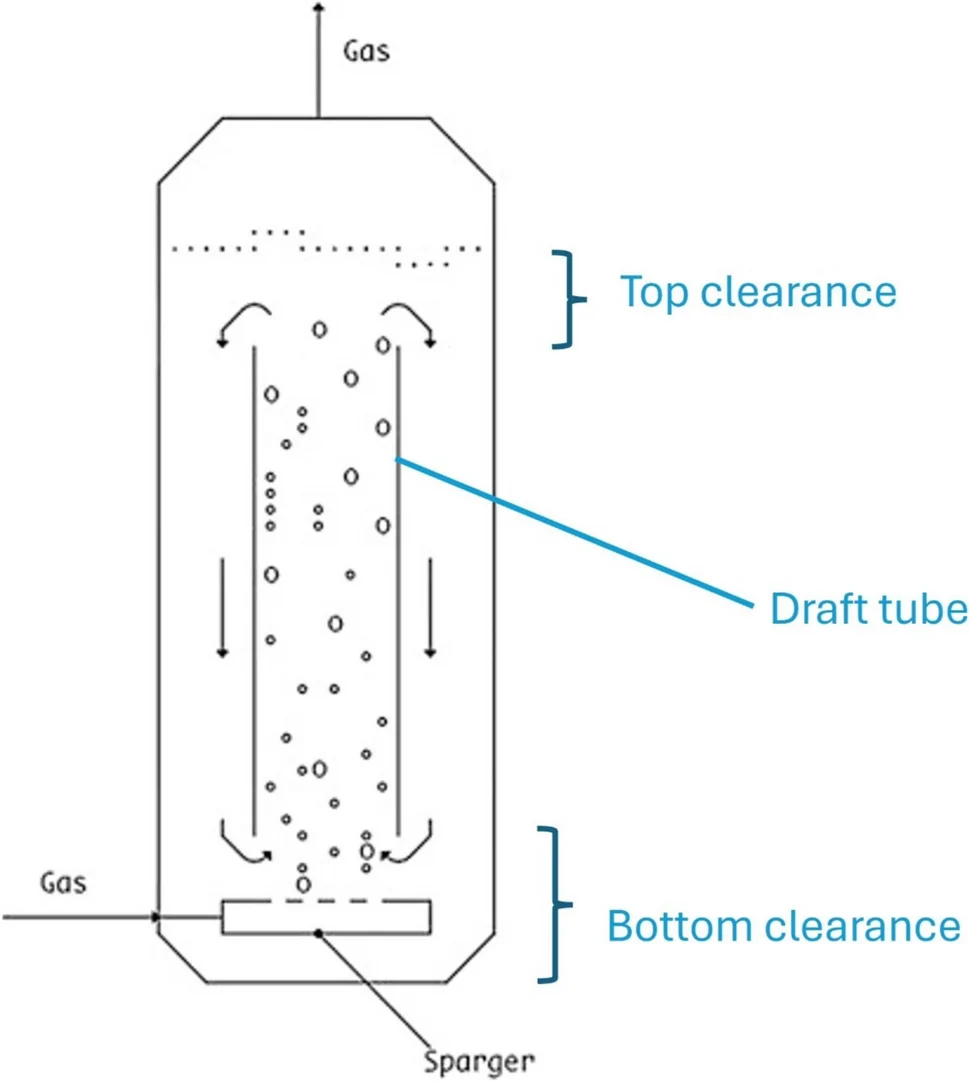
Airlift bioreactor with an internal draft tube. Picture adapted from Prado Barragan et al. (2016)

Airlift bioreactors are specialised equipment where the main recirculation motion of media and suspended biomass is promoted by the gas supply. These bioreactors are usually used for the cultivation of shear stress-prone microorganisms; the basic airlift bioreactor design is the bubble column bioreactor (Fig. 1) where the microbial biomass is suspended by a vertical motion of ascending bubbles. The simplicity of this design is mirrored by its economical appeal and its low energy requirements. Usually, these bioreactors are not useful for high cell density, but they are quite useful for recalcitrant compound biodegradation and for prolonged fermentation (Cerrone et al. 2011; Nili et al. 2022). A slightly more sophisticated design is the one that describes a proper airlift bioreactor, which is composed of a cylindrical vessel containing an internal (cylindrical) draft tube of smaller diameter (Fig. 2).

The gas is supplied through a sparger that is located at the bottom of the vessel. This configuration promotes strong fluid upward (in the riser) and downward (in the downcomer) motions, whose velocities are strictly driven by the constantly changing values of hydrostatic and hydrodynamic pressures (according to the Bernoulli law, Eq. (6), being *P*, pressure; *ρ*, fluid density; *V*, fluid velocity; *g*, gravitational acceleration; *h*, height of elevation; and *f*, a friction factor); the friction factor is given by the correlation between the Reynolds number and the sparger opening roughness, but in a situation where the turbulent Prandtl number equates to 1, it makes negligible the heat dissipation caused by friction at the point of gas sparging. The gas velocities are also variably dependent on the gas hold-up (*ε*), the diameter ratio of the vessel vs the draft tube (annular space), the height-to-diameter ratio of the vessel, and the top and bottom clearance (Fig. 2), together with the slip velocity of the bubbles, the changing bubble diameter, and the hydrodynamic behaviour of the individual air bubbles.

6 $$P+\frac{\rho }{2}{\mathrm{V}}^{2}+\rho \mathrm{gh}+{f}_{\mathrm{D}}=\mathrm{constant}$$

**Fig. 3:**
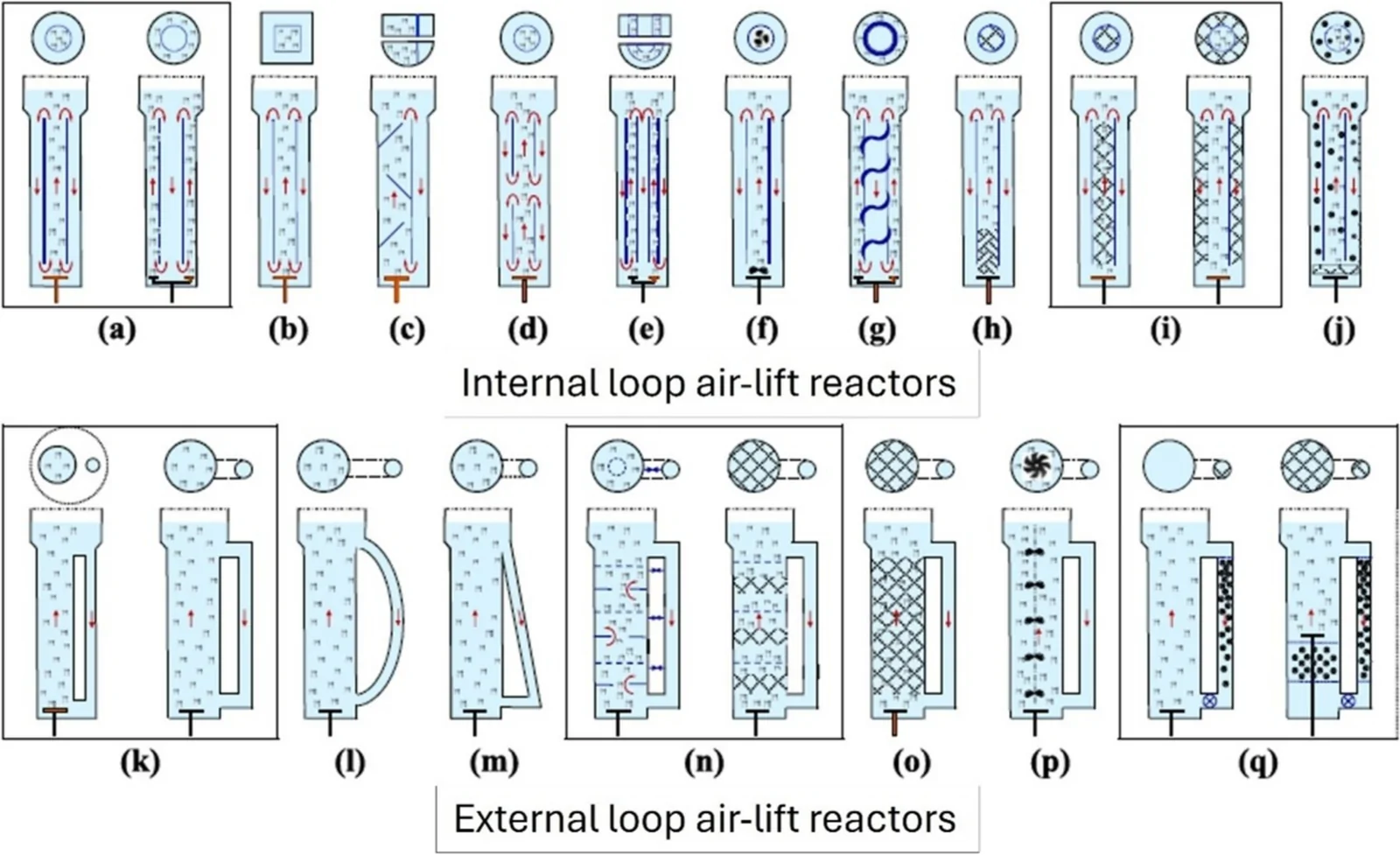
Airlift configurations as internal loop airlift reactor (ILALR) or external loop airlift reactor (ELALR). **a** Conventional (draft tube), **b** rectangular, **c** split-plate (rectangular/cylindrical/slanted baffles), **d** multistage draft tube, **e** multiple draft tube/multiple split-plate, **f** stirred, **g** helix, **h** static mixing, **i** packed bed, **j** fluidised bed (three-phase), **k** conventional, **l** hybrid D-shape, **m** hypotenuse/triangular, **n** multistage, **o** packed/fluidised bed, **p** stirred (several impellers), **q** inversed fluidised bed (tri-phasic). Picture adapted from Behin and Amiri (2023)

The areas of higher pressure drops (therefore with increased fluid acceleration) are located at the top exterior portion and at the bottom interior portion of the draft tube according to modelling made by Gavrilescu and Tudose (1998); these researchers identified a stronger contribution of the gas hold-up and of the gas superficial velocities in promoting stronger fluid recirculation; the gas hold-up (the total gas volume over the total vessel volume) is clearly different from the gassed portion (riser) to the ungassed portion (downcomer); this critical difference in fluid density, driven by the different gas hold-up, also affects buoyancy of the resuspended particles (solid pelletised filamentous fungal aggregates), but the effect on the fluid motion is usually exceeded by the gas superficial velocity (that also is interlinked with the fluid superficial velocity) (Camarasa et al. 2001). The slip velocity (the difference between the superficial velocity of the conveyed gas and the superficial velocity of the conveyed fluid particles) also affects the hydrodynamic behaviour of the fluid contained in the vessel. However, when suspended solid particles are contained within the vessel, the fluid dynamics of the system become more complex; the system is in effect a three-phase system. Olivieri et al. (2003) modelled the behaviour of suspended solids in these so-called three phases in an internal loop airlift reactor (ILALR). Their two main findings are as follows: (1) a direct correlation between the intensity of gas velocity and liquid circulation velocities, (2) the increased roughness of the suspended particles causes a reduction in turbulent flow at equal gas velocities (Olivieri et al. 2003). Garcia-Calvo et al. (1999) also tried to simplify the modelling by envisaging a three-phase airlift model that takes into account the differences in density of the resuspended solids when they differ from the liquid density; the main finding is that when the linear velocity in the riser exceeds the settling velocity of the resuspended particles, the airlift system is in circulating mode; when these two conditions are not met, the airlift system behaves progressively as a fluidised bed or as a packed bed. Zhang et al. (2021) analysed these phenomena specifically modelling pelletised fungal biomass (with an average size distribution of 2 mm) resuspended in a custom-made micro-fluidised bed (a sort of bubble column airlift); their main findings were that the packed density of pelletised fungal biomass greatly reduces a fluidised motion and that the vertical gas velocity is proportional to the pellets’ radial motion; the axial motion of the pellets is instead promoted by a turbulent flow. The basic design of the airlift bioreactor was altered by many variations for specific application purposes or simply to enhance heat and mass transfer and therefore increase the productivity of the system (Chen 1990). The traditional airlift configuration is shown in Fig. 3a; the two configurations shown in Fig. 3a differ only by the location of the air sparger (inside the draft tube or inside the annular ring, i.e. external to draft tube, where the draft tube is thus the downcomer). The principle dual difference in the variation of the traditional design is the location of the draft tube (internal or external) to facilitate an internal or external recirculation loop (Fig. 3a–j vs k–q).

**Fig. 4:**
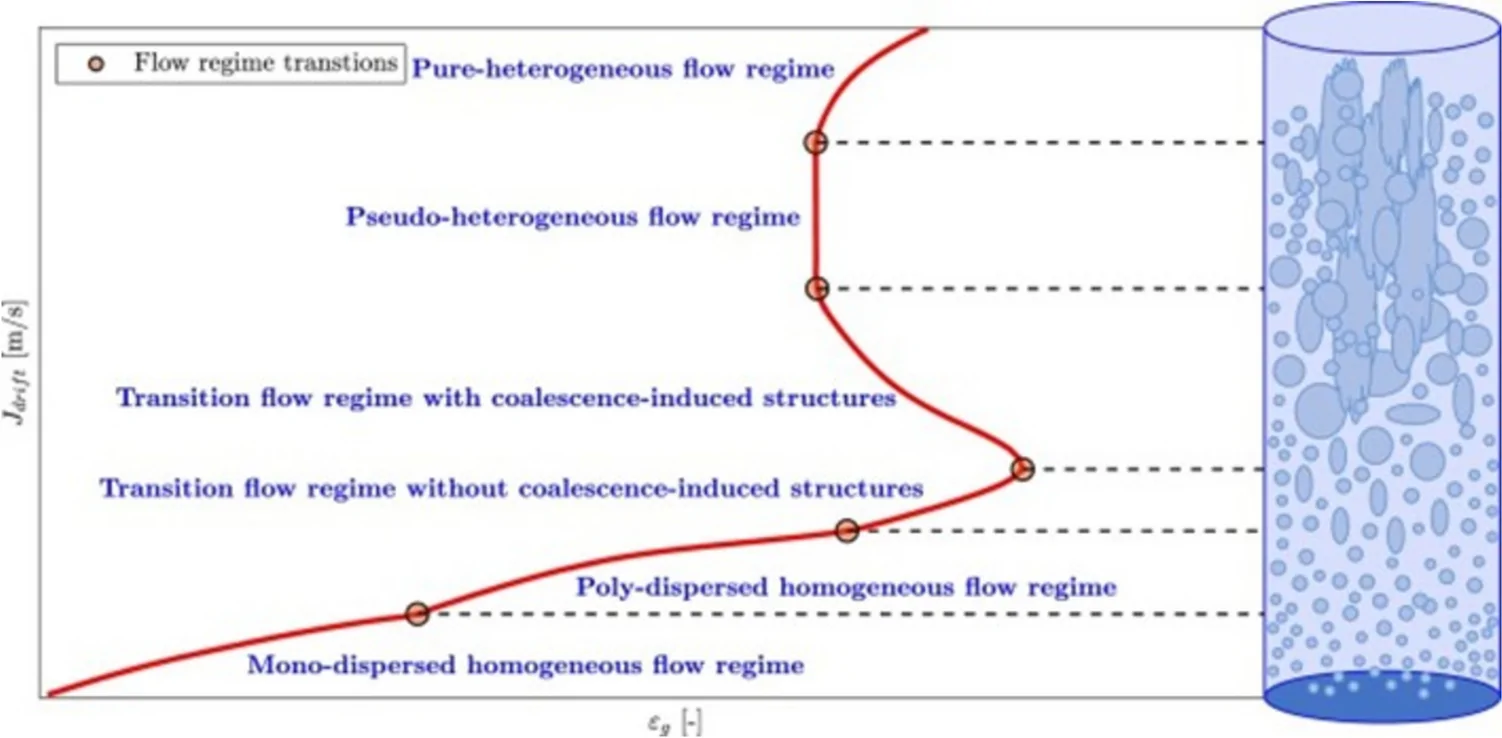
Correlation between the drift velocity (*J*_drift_, *m/s*) and gas hold-up (*ε*_g_) in establishing the gas flow and bubble dynamics behaviour in a sparged column. Picture adapted from Besagni (2021)

**Fig. 5:**
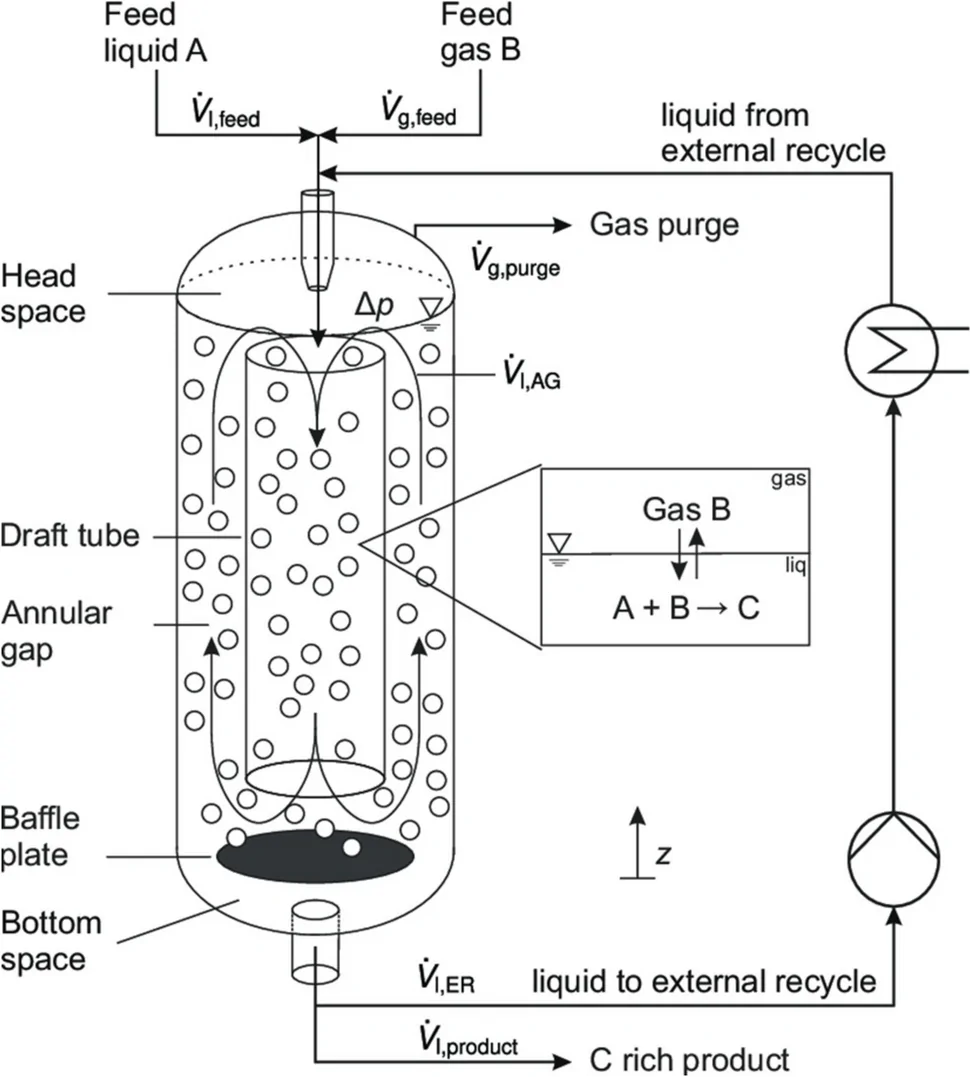
Jet-loop reactor schematic. Picture adapted from Breit et al. (2022)

The different configurations cause a variation in the hydrodynamic behaviours of the airlift bioreactors. There is a direct correlation between biomass volumetric productivity and oxygen availability in aerobic bioprocesses; oxygen availability is strictly dependent on the interfacial area of the sparged bubbles, bubble size distribution, rate of bubbles break-up, reduction of bubble coalescence, and increased gas hold-up (Chen 1990). Han et al. (2017) noticed that the gas hold-up (*ε*) is strictly dependent on the gas velocity, but when the sparger is in the annulus (draft tube) region, the gas hold-up is higher than when the sparger is in the central region of the riser. Han et al. (2018) also found that the foaming height of the medium is reduced if the air is sparged in the annular region; conversely, the liquid velocity is higher in the centrally sparged airlift compared to the annulus sparged airlift; in addition, there is a direct correlation between the increasing low gas superficial velocity (0.3 m/s to 1.18 cm/s) and the reduction of recirculation time; but this trend does not hold true at higher gas velocity (1.1 to 1.8 cm/s) where the higher gas hold-up in the downcomer becomes the driving force of the liquid recirculation. Smaller bubbles (< 0.1 mm) have slower rising velocity compared to the liquid velocity (slip velocity), and this factor also contributes to a higher gas hold-up (*ε*). Both the top clearance (degassing area) and bottom clearance (distance from the draft tube to the bottom of the vessel) of the internal loop airlift bioreactor are critically important for the efficient medium mixing and for the increase of volumetric oxygen mass transfer (K_L_a) to the bioreactor. When the top clearance is small (0–2 cm), the disengagement of the sparged bubbles from the liquid phase is suboptimal, and the recirculation of the media is severely reduced (even if the gas hold-up is high); when the top clearance is big (> than 6 cm), the disengagement is more efficient, but both the recirculation speed of the media and the gas hold-up decrease (Luo and Al-Dahhan 2010, Coimbra et al. 2024); on the other hand, when the bottom clearance is too small (< than 2 cm), the media recirculation is hindered by flow restrictions, while if it is bigger (> than 5 cm), the media recirculation improves but there are also increased chances of dead volume creation through backflowing eddies (Luo and Al-Dahhan,, 2010); both aspects become critical when we consider a three-phase system, where the suspended solids (mycelia aggregates) have the tendency to sediment. When the airlift bioreactor is provided with an external loop, the effect of the top clearance height over the gas hold-up and the media recirculation velocity are negligible (Liu et al. 2020). Another aspect that also dictates the variations in the design of the airlift bioreactor is the bubble dynamics in liquid media; this key aspect directly impacts on the sparger design and the geometrical dimensions of the bioreactor (Besagni 2021). Traditionally, the bubble dynamics and the consequential gas flow were directly linked to the gas velocity and the diameter vessel (Mendoza Martinez and Escamilla Silva 2013). Three major modes of gas flow were modelled: (1) slug flow (homogenous flow with a few big, coalesced bubbles), (2) churn-turbulent flow (inhomogeneous flow with multiple coalesced bubbles and a few small bubbles), (3) bubbly flow (homogenous flow with regularly sized fine bubbles). Besagni (2021) expanded this model, highlighting physical commonalities beyond the description of the behaviour of the gas flow; in this model, five transition regimes of gas flow were described (Fig. 4) by correlating the drift velocity and the gas hold-up; Besagni (2021) clearly highlighted that the sparger design (spider and/or perforated plate design with 0.5-mm openings and when used with low gas velocity. i.e. 0.03 m/s) promotes the ideal homogenous flow regime with narrow bubble size distribution (3 mm, on average). Outside of these constraints (for example with a higher gas velocity) and without anti-coalescent molecules, a heterogenous gas flow is likely. The dynamic change of the bubble diameter after leaving the sparger is directly correlated with the sparger design, with the potential of coalescence phenomena between bubbles and with the control of the drag and lift forces over the individual bubbles (Rollbusch et al. 2015); in this context, a series of unitless number are used as useful tools for modelling (Eötvös, Morton and Bond numbers); these numbers correlate the ratio of the fluid density and viscosity with the surface tension of the bubbles. Usually, the critical bubble diameter is interlinked to the bubble rising behaviour (being intimately linked to the bubble morphology, spherical vs ovoidal) (Ziegenhein and Lucas 2019); these authors suggest a specific bubble diameter (5.1 mm) where this morphology change occurs; the increasing uprising velocity of the airflow is directly correlated with the increasing bubble diameter above a critical 2-mm size; this effect is caused by a swelling effect driven by the hydrostatic pressure (Fan et al. 2023); due to the complexity and of the interplaying of different phenomena, such as the presence of drag/lift forces, a turbulent regime, and a three-phase system, the direct correlation of the increased uprising velocity with the bubble diameter can be altered (detailed descriptions of these effects are not the object of this review; therefore, the authors redirect the reader to the following publications (Rollbusch et al. 2015) (Li et al. 2018) (Ziegenhein and Lucas 2019)). A highly efficient variation of the airlift bioreactor is called the jet-loop reactor (Fig. 5); the main feature of this reactor is the presence of downward pointing nozzle that mixes the liquid and the sparged gas; it has been used to promote biodegradation of recalcitrant compounds (Eusebio et al. 2004; Mugaishudeen et al. 2024) or to promote chemical reactions, such as hydration (Li et al. 2024). An efficient design of a jet-loop reactor (Buss Loop Reactor®) has been manufactured by Buss ChemTech AG™, and it shows a remarkable volumetric oxygen mass transfer (K_L_a of 207 h^−1^); an even higher volumetric oxygen mass transfer (up to a K_L_a of 2250 h^−1^, with expected 610 g/h biomass formation rates) has been highlighted by Weber et al. (2018); these solutions, if the power input (P/V) supports the economic justification, can be ideal for process intensification, but to the best of our knowledge, these configurations have never been used for cultivation of filamentous fungi; presumably, they could be used efficiently for bacterial cultivation (Ughetti et al. 2018).

These hydrodynamic considerations regarding a two-phase system (gas/liquid) are modelled by computation fluid dynamics (CFD) with a sufficient accuracy, but the presence of suspended solids alters greatly the internal dynamics, and it can affect the evolution of the bioprocess with somehow unpredictable outcomes. A pelletised mycelial submerged cultivation is a classic three-phase system, and it can be compared to a fluidised-bed reactor where the suspended solids are pelletised aggregates of mycelia (Zhang et al. 2021); the efficient fluidisation motion is maintained, provided that the pellet size distribution is controllable and uniform (Zhang et al 2021). Despite the complexity of modelling and operating a three-phase system of an airlift bioreactor, one of the key advantages of this type of bioreactor (regarding its scaling considerations) is that the liquid recirculation rate and the liquid recirculation time can be kept basically constant independently of the scale (Chen 1990); on the contrary, mechanically agitated bioreactors decrease in efficiency through scaling, creating wide areas of suboptimal media mixing (Chen 1990; Losoi et al. 2022), and they also require higher power inputs to operate.

## Airlift bioreactor cultivation of filamentous fungi: advantages and disadvantages

Airlift bioreactors are one of the preferred options for the cultivation of filamentous fungi; the main advantage of using this set-up is the prevention of the shear stress that these microorganisms might encounter when mechanically agitated in a stirred tank bioreactor (STR). The major features of an airlift bioreactor are equally responsible for its advantages and for its disadvantages. The main advantages of using airlift bioreactors for the cultivation of filamentous fungi are (1) lower power consumption, (2) reduced shear stress, (3) excellent fluidisation of the system, (4) simple morphology, and (5) reduced internal surface area that could promote biomass adhesion. The main disadvantages are (1) suboptimal volumetric oxygen mass transfer (K_L_a) in some cases, (2) potential reduction of nutrient mixing (and therefore creation of nutrients gradients and potential suboptimal biomass yields), (3) higher foaming potential, and (4) lack of biomass homogenisation by a mechanical stirring that could be potentially a detrimental factor. Each feature either promotes or hinders the optimal potential of the bioreactor for the cultivation of filamentous fungi, and often, these characteristics are interacting for a combined synergistic or antagonistic outcome.

### Airlift cultivation of filamentous fungi for the production of released products

As mentioned, the airlift-based cultivation of filamentous fungi is the preferred approach due to the reduced shear stress the microorganisms face with the absence of a mechanical agitation. The medium cell densities achieved with this strategy are particularly suited for the prolonged cultivation of filamentous fungi in continuous fermentation (Cerrone et al. 2024). Filamentous fungi of both *Basidiomycota* and *Ascomycota* divisions are cultivated in submerged fermentation because they can produce a variety of organic acids and enzymes that find applications in the food industry sector, in the biomedical sector, in the bioremediation sector, and in the biorefining sector. A few industrially relevant filamentous fungi are particularly recurring because they are established microbial workhorses for biobased manufacturing and for enzyme production; among the most recurring genera are *Aspergillus* sp., *Penicillium* sp., *Trametes* sp*.*, *Pycnoporus* sp., *Trichoderma* sp., *Neurospora* sp., *Fusarium* sp., *Coprinus* sp., and *Mucor* sp. Some of them are so well established in the bioprocessing sector that they have gained the role of model organisms (Brandl and Andersen 2017). A pioneering study by Träger et al. (1989) cultivated *Aspergillus* sp. in batch fermentation using a custom-made ELALR (with a working volume of 260 L) with low biomass production (Max 3.7 g/L) but with titres of gluconic acid up to 81.1 g/L after 19 h. Another pioneering study by Park et al. (1994) produced itaconic acid by cultivation of *Aspergillus terreus* in repeated batch strategy in ALR with an average biomass concentration of 12 g/L; the product yield was 0.48 g/g for a duration of up to 21 days. The airlift bioreactor is well suited for the fungal biodegradation of recalcitrant compounds (polyaromatics, benzene-derivatives, and a variety of organic dyes); this happens through the activation and release of fungal enzymes, particularly laccase, lignin peroxidase, and Mn-peroxidase (Teerapatsakul et al. 2017; Zhuo and Fan 2021); Sodaneath et al. (2017) utilised a pure culture of *Bjerkandera adusta* to decolorise three acidic dyes within 15 h of treatment for an efficiency up to 90%. Souza et al. (2014) used a three-*Agaricomycetes* inocula for the partially efficient COD reduction/colour removal and concomitant phenol biodegradation in an airlift bioreactor. Michelin et al. (2013) compared stirred tank bioreactors and airlift bioreactors and concluded that at the same K_L_a, the airlift set-up is more efficient for the production of xylanase and β-glucosidase in batch mode using 1% of corncob as carbon source for 15 days (7000 and 2100 U/L, respectively); a higher amount of cellulase and a slightly lower amount of xylanase were produced by Todero Ritter et al. (2013) with an intensification process (fed-batch) using *Penicillium echinulatum* (6400 and 5500 U/L, within 100 h and 64 h, respectively*)*. A pectinase activity of two orders of magnitude higher (1 × 10^5^ U/L) was obtained in STR and ELALR by Fontana and Moura da Silveira (2012) cultivating *Aspergillus oryzae* for batches of 42–45 h with a slightly advantageous performance by the STR (12% higher); the same authors also compared STR and ILALR for the production of extra or intracellular pectinase, highlighting that ILALR produced 30% higher specific enzymatic activity, using the same media (Fontana et al. 2009). Liu et al. (2013) utilised a 65-L airlift bioreactor to cultivate *Pycnoporus* sp. for the production of laccase up to 72,000 U/L after 6 days of cultivation, showing both a similar yield and specific enzymatic activity (Fontana et al. 2009). All the previously mentioned studies highlight the advantage of using an airlift bioreactor for enzyme production when cultivating filamentous fungi; the main reason for this is the gentle pneumatic motion promoted by the ALR. The direct comparison of fungal production of enzymes with ALR or STR is a particularly useful benchmark when comparing methods of growth of filamentous fungi at scale; Galahup et al. (2002) used a 15-L STR growing *T. pubescens* with a constant 0.06 g/L/h glucose feed (below inhibiting level), which was able to produce 743,000 U/L for a specific laccase yield of 67 U/g (i.e. sixfold higher than every previously mentioned study in this review, using ALR). The reasons behind this superior performance regarding the enzyme production are attributable to two causes: the addition of CuSO_4_·5H_2_O, and the prolonged duration of the fermentation (sevenfold longer than the best result with ALR reported by other studies); the authors highlight the fact that the increased laccase production only happened when the fungal biomass reached a steady-state (after 300 h); it is therefore worthy to noticing that a prolonged ALR fermentation could also have achieved similar results, considering that the specific enzyme production at the same timepoint was comparable in both systems. The mechanical agitation does not seem to induce a particular advantage in the laccase production when cultivating filamentous fungi; therefore, it can be ascertained that the ALR is also a suitable platform for enzyme production by filamentous fungi (Liu et al. 2013). Filamentous fungi have evolved their proteic machinery so as to be particularly efficient in biodegrading lignocellulosic materials; for this reason, they produce not only laccase but also lignin peroxidase and manganese peroxidases (Herpoël et al. 1999; Shi et al. 2021). Increasing volumetric productivities can be achieved in controlled environments such as bioreactor settings; for this reason, different researchers scaled up the methods of enzyme production, either cultivating filamentous fungi in stirred tank reactors (STR) (Nüske et al. 2002) or in airlift bioreactors (ALR) (Bonnarme et al. 1993). Both bioreactors set-up showed a comparable specific enzymatic activity for both manganese and lignin peroxidase (MAX 835 U g^−1^ for STR and 5076 U g^−1^ for ALR). It can be argued that the likely increased K_L_a obtained in the STR, responsible for 92% higher biomass concentration, overcomes the lower enzyme production in the ALR, which resulted in higher enzymatic volumetric productivities (19 U L^−1^ h^−1^ (ALR) and 44 U L^−1^ h^−1^ (STR)). Due to this lignocellulosic biodegradative potential, these enzymes are especially apt for the biodegradation of phenol-rich agricultural wastewaters; Olivieri et al. (2012) demonstrate a highly successful polyphenol biodegradation by a continuous fermentation in an airlift bioreactor; their approach was to perform repeated batches to maximise the phenol biodegradation (up to 68%) through a continuous fermentation; with this strategy, they were able to extend the fermentation up to 250 h. Most of the filamentous fungi prefer a spreading behaviour to promote hyphal interaction and overlapping (where sexual mating is one of the reasons); therefore, it is not surprising that they prefer to adhere to solid substrates. One of the potential advantages of cultivating filamentous fungi for the production of extracellular products is that the bioreactor set-up could also include a solid support, when this feature does not hinder the convenient separation of the metabolites of interest and if the solid support does not harm the physiology of the microorganisms; to the best of our knowledge, very few researchers adopted the approach of using a full solid-state fermentation in an airlift bioreactor (Gonzalez-Ramirez et al. 2014 and Sierra-Solache et al. 2016), possibly because this set-up likely causes the loss of the pneumatic motion; but many other researchers used instead a similar approach, opting for a fluidised-bed bioreactor (FBB). A fluidised-bed bioreactor merges the advantages of the adhesion to a solid support; in this instance, the solids of support (for example, made of alginate or wheat-coated polystyrene) (Lu et al. 2020) can be added to promote mycelia adhesion; on the other hand, the main suspended solids can be the pelletised mycelia themselves (by self-aggregation phenomena). Cruz-Morató et al. (2013) used an FBB for the biodegradation of pharmaceutical compounds with *T. versicolor* as the biocatalyst; different success of removal of up to 75% of these emerging pollutants was achieved. Dalecka et al. (2021) used a dual inocula (*Trametes* sp. and *Aspergillus* sp.) to remove ibuprofen, ketoprofen, and metoprolol in a pelletised FBB with > 90% efficiency. All the above-mentioned studies that adopted the cultivation of fungi in ALR have been demonstrated to be suitable for both enzyme production and chemical production, e.g. organic acids. When the focus is on the biodegradative performance, aiming at medium/high cell densities is not necessary and is more important to achieve a prolonged fermentation; for this reason, the use of ALR for submerged cultivation of fungal biomass is meritory.

### Airlift cultivation of filamentous fungi for the production of biomass

The use of ALR to cultivate filamentous fungi has some intrinsic challenges when the fungal biomass is the targeted product of the bioprocess. Very few studies used ALR for the direct production of fungal biomass. The control of the biomass pelletisation for the fluidification of the filamentous fungi becomes challenging when one wants to maximise the biomass volumetric productivity and therefore achieve medium or high cell densities (Cairns et al. 2019a); multiple phenomena of localised adhesion of biomass are likely on all surfaces of the bioreactor. The presence of multiple surfaces in a bioreactor increases proportionally the possibilities of adhesion (Waldherr et al. 2023). A STR, having a central rotating shaft with baffles, also provides multiple locations of adhesion for mycelia (Kelly et al. 2006; Buffo et al. 2020; Sanchez-Vargas et al. 2024). On the other hand, a STR has a higher potential to reduce the volume of each pellet of fungal biomass (Waldherr et al. 2023). ALR has a reduced surface area for adhesion, because of the lack of an internal stirrer; but the lack of mechanical disruption means the reduction of the volume of each aggregating pellet is hindered (Cerrone et al. 2024). Bubble column bioreactors (BCB) have the least surface exposure for adhesion but suffer from the drawback of having reduced biomass concentration and reduced volumetric biomass productivities (Quaratino et al. 2006). ALR and STR can be compared in terms of biomass volumetric productivities because the prolonged fermentation that can be obtained in continuous feeding mode using ALR induces volumetric productivities in the range of 0.5–0.87 g/L/h (Cerrone et al. 2024), while STR can be repaid by volumetric productivities of 0.25–0.64 g/L/h (Jüsten et al. 1998; Khalesi et al. 2014; Bakratsas et al. 2023). Many studies adopted ALR for the cultivation of filamentous fungi at medium/high cell densities, where the fungal biomass was the targeted product. Jung et al. (2006) grew *H. erinaceus* in a 3-L ALR to get the highest biomass concentration of 14 g/L after a 5-day batch and for a biomass yield of 0.65 g/g; those titres and yields were comparable with STR batch cultivations done by the same authors up to a working volume of 500 L. Many studies preferred to cultivate filamentous fungi in ALR in batch mode; the likely reason for this is the simplicity of the design of experiments (Table 1). A number of studies achieved high biomass with Souza Filho et al. (2017) producing up to 65.47 g/L of *Rhizopus oryzae* in an undiluted potato protein liquor (with 120 g/L of total sugars and with 300 g/L of total COD). Nair et al. (2018) used lignocellulosic side streams in an integrated cascading bioprocess to grow filamentous fungi in ALR (*N. intermedia*) and produce bioethanol as a downstream product; the maximum mycelia biomass was 21.4 g/L after 120 h. Choi et al. (2011) grew *Mycoleptodonoides aitchisonii* for a maximum biomass concentration of 20.3 g/L after 14 days of cultivation in a 5-L ALR in rich media with lactose as a C source. A few studies have scaled the growth of filamentous fungi beyond the typical 1- to 5-L reactors (Table 1); some were for extracellular product production, and of those that focused on biomass production, a few are worth highlighting; Furlan et al. (2024) reported on an ambitious study using a 20% v/v corn steep liquor (CSL) medium for the cultivation of *Rhizopus microsporus* (variety *oligosporus*) grown in a 12.7-L ELALR; the maximum achieved biomass was 38.3 g/L after 96 h, with a projected dry biomass production value of 46.4 kg from a 1-m^3^ ELALR. Chiang and Chiang (2013) cultivated *Antrodia cinnamonea* in a 500-L ALR to a maximum of 5.4 g/L of fungal biomass with a chemically defined media where glucose was the main carbon source, for a total batch fermentation of 42 days.

**Table 1:** Resuming table reporting results from scientific studies using bioreactors to produce released products (organic acids/soluble enzymes) cultivating filamentous fungi (top section). Resuming table reporting results from scientific studies using bioreactors (ALR) for the production of fungal biomass through submerged cultivation (bottom section)

Volume (L)	Biomass concentration (g/L)	Product concentration (g/L or *U/L)	Product yield (g/g or **U/g)	Volumetric productivity (g/L/h)	Mode	Platform	Strain	Duration (days)	Authors
260	3.7	81.1	0.811	4.15	Batch	ELALR	*Aspergillus*	0.81	Träger et al. (1989)
2	12	48	0.48	0.37	Repeated batch	ALR	*Aspergillus*	21	Park et al. (1994)
2	7.5	N/A	N/A	0.375	Batch	ALR	*Bjerkandera*	0.83	Sodaneath et al. (2017)
0.35		80	N/A	N/A	Batch	ALR	*Agaricomycetes*		Souza et al. (2014)
9.5		*7100/2100	N/A	N/A	Batch	STR/ALR	*Aspergillus*	15	Michelin et al. (2013)
3.5	5.1	*6400/5500	**1078.43	N/A	Fed-batch	ALR	*Penicillium*	4.16	Todero Ritter et al. (2013)
4.1	10	*149,000	**14,900	0.22	Batch	ILALR/ELALR	*Aspergillus*	1.87	Fontana et al. (2012)
3.1	10.1	*60,600	**6000	0.10	Batch	ILALR/STR	*Aspergillus*	4	Fontana et al. (2009)
65	6	*72,000	**12,000	0.08	Batch	ALR	*Pycnoporus*	6	Liu et al. (2013)
15	11.1	*743,000	**66,936.93	0.03	Fed-batch	STR	*Trametes*	12.5	Galahup et al. (2002)
2.2	2.7	*4500	**1666.66	0.05	Batch	STR/ALR	*Phanerochaete*	4.16	Bonnarme et al. (1993)
7	0.2	*2500	**12,500	0.02	Continuous	ALR	*Pleurotus*	10.41	Olivieri et al. (2012)
1.5	2.533333333	*300	**118.42	0.03	Continuous	FBB	*Trametes*	4	Cruz-Morató et al. (2013)
1.25	N/A	N/A	N/A	N/A	Batch	FBB	*Trametes/Aspergillus*	28	Dalecka et al. (2021)
**Volume (L)**	**Biomass concentration (g/L)**	**Product concentration (g/L)**	**Product yield (g/g)**	**volumetric productivity (g/L/h)**	**Mode**	**Platform**	**Strain**	**Duration (days)**	**Authors**
3	14	14	0.65	0.04	Batch	ALR	*Hericium*	5	Jung et al. (2006)
5	13.4	13.4	0.44	0.07	Batch	ALR	*Paecilomyces*	8	Xu et al. (2006)
5	10.4	10.4	0.34	0.04	Batch	ALR	*Fomitopsis*	10	Choi et al. (2007)
5	20.3	20.3	0.33	0.06	Batch	ALR	*Mycoleptodonoides*	14	Choi et al. (2011)
500	5.4	5.4	0.18	0.01	Batch	ALR	*Antrodia*	42	Chiang and Chiang (2013)
2.5	7.04	7.04	0.13	0.10	Batch	ALR	*Rhizopus*	3	Nitayavardhana et al. (2013)
4	65.4	65.4	0.51	1.21	Batch	ALR	*Rhizopus*	2.25	Souza Filho et al. (2017)
4	4.35	4.35	0.28	0.09	Batch	ALR	*Rhizopus/Mucor*	2	Satari et al. (2016)
3.5	10.17	10.17	0.54	0.21	Batch	ALR	*Aspergillus/Rhizopus/Mucor*	2	Asadollahzadeh et al. (2018)
3.5	21.4	21.4	0.30	0.18	Batch	ALR	*Neurospora*	5	Nair et al. (2018)
14	38.3	38.3	0.47	0.40	Batch	ELALR	*Rhizopus*	4	Furlan et al. (2024)
160	8.5	8.5	0.46	0.85	Semi-continuous	ILALR	*Aspergillus/Rhizopus*	3	Jin et al. (2001)
4	13.4	13.4	0.23	0.87	Semi-continuous	ILALR	*Trametes*	2.95	Cerrone et al. (2024)

Satari et al. (2016) opted to use two strains (*Rhizopus oryzae* and *Mucor indicus*) in a 4-L ALR cultivation using invertase-treated citrus waste as substrate; the maximum fungal biomass concentration was 4.35 g/L with a yield of 0.28 g of biomass/g of total sugars. Asadollahzadeh et al. (2018) cultivated three different strains of filamentous fungi (*Aspergillus oryzae*, *Mucor indicus*, and *Rhizopus oryzae*) achieving 10, 6, and 5 g/L of biomass concentration, respectively, in a 60% diluted spent sulphite liquor media in ALR. To the best of our knowledge, only two studies focused on a (semi) or continuous fermentation strategy using an ALR for the cultivation of filamentous fungi (using different strains). These studies focused on the cultivation of filamentous fungi as producers of mycoproteins growing them in chemically defined media or in organic wastewaters. Jin et al. (2001) cultivated *Aspergillus* and *Rhizopus* sp. up to 8.5 g/L through a semi-continuous fermentation strategy in ELALR of 160 L from a starchy wastewater substrate; Cerrone et al. (2024) grew *Trametes versicolor* in a 4-L ILALR using a CSL-based media with a starting concentration of 10 g/L of glucose and with subsequent semi-continuous feeding regime using the same media; the achieved production was total maximum biomass (CDW) of 13.4 g/L using a dilution rate of 0.02 h^−1^ and with a maximum volumetric productivity of 0.87 g/L/h at a dilution rate of 0.1 h^−1^. Semi-continuous cultivation of filamentous fungi in ALR imposes specific challenges if one wants to accurately control the dilution rate by concomitantly removing the spent media and part of the produced biomass. This is especially critical at high dilution rates, where the residence time is much shorter. The filamentous fungi have slower doubling times with respect to bacteria (three- to fourfold slower) so the risk of biomass wash-out is much higher; filamentous fungi induce a localised variation of nutrient uptake due to the mycelia aggregations; this partially uncontrolled behaviour increases the difficulties in stopping biomass adhesion; this feature becomes critical when ALR are specifically used for filamentous fungi biomass production.

Despite the differences in performance in the above-mentioned studies, almost all the studies stated that the hydrodynamic airlift regime is lost above a critical threshold concentration of biomass due to the increased volume of individual pellets and the clumping phenomena; this dynamic phenomenon converts an initial Newtonian fluid into a pseudoplastic non-Newtonian fluid where adhesion phenomena are highly likely; the cascading effect impacts on the overall oxygen availability and in nutrient gradient formation. Selected fungal strains capable of maintaining micro-pellet morphologies can be envisaged as preferred for promoting a prolonged duration of the fermentation, especially if the fluidised motion can be homogenously maintained. A range of controls can be envisaged so to promote the control of a homogenously micro-pelletised mycelia population. These features could be enacted in combination or individually, and they are inoculum density, physico-chemical control of the pellet size, hydrodynamic regimes, and bioreactor design. Engineering aspects of different bioreactor designs, in the context of fungal biomass aggregation, will be dealt later (“Hydrodynamics challenges for the submerged cultivation of filamentous fungi in airlift bioreactors” section of this review).

## Effect of the inoculum density on the successful cultivation of filamentous fungi in airlift bioreactors

The peculiarity of the filamentous growth of hyphal cells is directly related to the resulting pellet density, pellet volume, and pellet porosity when grown in submerged fermentation in ALR. The binary fission of non-filamentous bacteria and their exponential population growth can be modelled by the following simple Eq. (7) used to calculate the doubling time (*G*).

7 $$\mathrm{G}=\frac{\mathrm{t}}{3.3{\mathrm{logN}}_{\mathrm{t}}/{\mathrm{N}}_{0}}$$

The equation is used for the generation (doubling) time calculation, where *t* is the timeframe considered during the exponential growth phase, *N*_t_ is the final amount of the number of cells at the end of the exponential growth phase, and *N*_0_ is the initial amount of number of cells at the beginning of the exponential growth phase.

The non-filamentous bacterial population, following this equation, would produce a homogenous population of increasing density, as long as the nutrients and oxygen to sustain the growth are available. Some specific (predatory) bacteria can adapt their binary fission in response to the environmental conditions (Plaşkowska et al. 2023). Regarding the growth of filamentous fungi, the cubic root model mentioned earlier can model the growth of filamentous hyphal cells, pellet formation, and their consequential volume increase. Equations (1) and (2) correlate directly the oxygen availability as a driver for pellet formation. The mechanical disruption of the pelletised mycelia is one of the most useful methods for the creation of nucleating microaggregates of hyphal cells (Zweck et al. 1978). Similarly, the mechanical disruption of the fungal biomass in an STR has been explored as the key linearly correlating factor between the pellet size and the stirrer speed. Assuming a fractal behaviour of the hyphal aggregates and assuming an inelastic behaviour of the hyphal cell wall, these two features can be correlated to its decreasing thickening from the actively growing tip (Heydarian et al. 2000). The vegetative growth of mycelia aggregates in submerged cultivation excludes the possibility to obtain basidiospores, which is only produced by basidiocarps; these basidiocarps are only produced by specific environmental conditions in the wild or in controlled mushroom cultivation facilities; basidiospores have to be collected by a highly efficient method that controls the atmospheric mist concentration (Rockett and Kramer 1974a); a successful collection of basidiospores is the most accurate method of establishing an exact inoculum size (Rockett and Kramer 1974b). *Ascomycota* can instead produce asexual spores (conidia) under laboratory conditions, ruled by specific photonic and genetic mechanisms (Park and Yu 2012). Knowing the spore concentration in the inoculum can directly correlate with the resulting population density in the culture. Papagianni (2006) linearly correlates the pellet morphology and the pellet density, assuming that the hyphal aggregates are governed by a fractal behaviour; spore concentration of 10^3^–10^5^/mL produces pellets, while 10^6^–10^7^/mL produces clumps/aggregates and 10^8^–10^9^/mL produces dense homogenous filamentous hyphal cells, with a linear morphology. Because of the correlation between pellet morphology and productivity (Veiter et al. 2018), many researchers attempted to understand the evolution of the pellet size and the link with the inoculum density in the submerged cultivation of fungal biomass. Jones et al. (1993) linearly correlated a medium shear stress (16,666 RPM) applied for 30 s with the most reproducible narrow frequency distribution (pellet size of 100 µm^2^) for a fractal fragmentation (*D* = 0.76). Müller et al. (2022) performed a comprehensive study where they correlated the size distributions, area fractions of spores, spore agglomerates, spore agglomerates within pellets, pellets size, and dispersed mycelia by a high-throughput stereomicroscopy approach that tracked live cells imaging; their main finding is the inverse correlation between the decrease of spore concentration from 10^5^ to 10^3^ spore/mL and the increasing pellet size from 250 to 450 µm in the first 27 h of growth, followed by a stabilisation of both values. According to Liao et al. (2007), the increasing spore concentration did not affect the morphology of pellets of *Rhizopus oryzae*, but it is directly correlated to the pellet number and biomass concentration and inversely correlated to the pellet’s diameter. A similar effect was seen by Zhang et al. (2023) with cultures of *Monascus* sp. To the best of our knowledge, there are no studies that specifically focus either on the density of the mycelia fragments or spore concentration for the understanding of the pellet density in the ALR; the drivers behind pellet formation are well known as mainly linked to hydrophobic and electrostatic interactions between mycelial aggregates (Zhang and Zhang 2015). An analogous phenomenon to link inoculum concentration and resulting biomass density can be driven by the study conducted by Ansari et al. (2019) for the cultivation of microalgal pellets; in this study, the controlling factor for the formation of pellets of filamentous *Cyanobacteria* is light. A medium inoculation density (900 mg/L) allowed the highest biomass production (180 g/L) and the highest exopolysaccharide concentration; hydrophobic interactions of the filamentous cells promote cell aggregation, and there is a direct proportional correlation between inoculum density and final biomass production. Mechanical disruption by a submerged homogeniser has been also demonstrated as the most effective method for downstream extraction of bioactive from fungal biomass (Taubert et al. 2000), but a systematic understanding of the mechanical disruption and its correlation with the inocula size density and its implication for fungal cells cultivation has been less explored.

## Control of the pellet size and morphology for the successful cultivation of filamentous fungi in airlift bioreactors

Different drivers are involved in controlling the fungal pellet formation in ALR, and this is linked with the ability to prolong the duration of the ALR-based fermentation. Park et al. (1994) highlighted that a strategy of repeated batches (without cell recycling) in ALR allowed them to prolong the production cycle up to 21 days, at the expense of reduced product volumetric productivity of 0.37 g/L/h but with the capacity to achieve an average biomass concentration of 12 g/L. Blanquez et al. (2006) successfully focused on a semi-continuous strategy that targeted a 1/3 biomass removal every 21 days, from a steady-state biomass that had a relatively low concentration (2.3 g/L); with this approach, the researchers were able to prolong the ALR-based fermentation up to 91 days before the airlift motion was lost due to clumping. Jin et al. (2021) used an STR instead and highlighted the following findings: the frequency of mechanical disruption correlates with *Aspergillus* sp. pellet size and the citric acid production rate; the researchers saw a direct relationship between the increased frequency of mechanical disruption and both the pellet size decrease and the pellet concentration; the prolonged mechanical disruption and the cell retention (with a 70% of recycling rate) allowed the increased production of citric acid up to 193 g/L; even if this study focused on semi-continuous fermentation by using an STR, it highlights the need of pellet size control (ideally down to mycelia fragments) for an increased bioprocess productivity and for the enhancement of the fungal biomass density in semi-continuous fermentation. Nair et al. (2016) identified the pH (3.0–4.0) of the cultivation media as a key factor to control the pellet size of the biomass of *Neurospora intermedia* in ALR and avoid the formation of dispersed mycelia. The same effect was seen by Hernandez-Cruz et al. (2024) cultivating *Basidiomycota* and *Mucoromycota*. Cairns et al. (2019b) used a high-throughput automated live imaging tracking approach to infer the heterogeneity of the pellet size distribution and the ratio of dispersed mycelia vs pelletised aggregates. They linearly correlated the hyphal length and branch rate with the pellet diameter. In addition to that, they also reduced the expression of a γ-adaptin gene (*aplD*), and they noticed that this feature induces a hyperbranched morphology of the hyphal cells. The control of the hyphal morphology and the pelletising potential is a route that was explored by some researchers; Miyazawa et al. (2020) directly correlate the presence of α−1,3-glucans synthesis in the outer layer of the hyphal cells and the pelletised morphology; this correlation is so strict that the pelletised morphology is directly dependent on the percentage of α−1,3-glucans; conversely, when the α−1,3-glucans is synthesised as a component of the inner layer, the hyphal morphology is loose. A similar effect is seen with the synthesis and percentage increase of galactosaminogalactan (GAG), especially with a high degree of deacetylation. A mutant *A. oryzae* with an impaired α−1,3-glucans and GAG synthase activity (AGΔ-GAGΔ) produces hyphal cells only capable of loose aggregation and no pelletised formation. This feature is controversial, because α-1,3-glucans are usually one of the sought-after products of submerged cultivation of fungi; therefore impairing their biosynthesis in order to maximise biomass productivity could be a detrimental feature. Probably, a reversible control of this specific genic expression could be an option to not decrease the volumetric productivity. Fitz et al. (2019) deleted a key gene for GTPase production (*rac1*) in *Trichoderma reesei*; these researchers noticed that this factor increased the hyperbranching of the mycelia; this feature promotes a stronger pelletised morphology and causes a loss of hyphal polarisation. A rational approach that combines the control of the expression of key genes involved in the filamentous morphology and/or the hyphal branching/hyphal polarisation with a high-throughput automated micro-computed tomography can be a powerful tool to have an understanding of pellet evolution in the submerged cultivation of fungi (Meyer et al. 2021).

A less sophisticated approach than genetic control, it is the addition of specific chemicals in the cultivation media that control the pelletised morphology of the fungal cells in submerged fermentation. These research lines go in three directions: addition of microparticles, addition of surfactants, and addition of salts. Antecka et al. (2016) focused on microparticles (Al_2_O_3_) enhanced cultivation (MPEC) of *Pleurotus sapidus* and *Cerrena unicolor*, a decreased pellet diameter was clearly seen with a concentration of 15 g/L of Al_2_O_3_, and this factor is linked to a higher enzymatic productivity, likely due to better oxygen availability. Kurakake et al. (2017) adopted nonionic surfactants for the reduction of pellet diameters of *Aspergillus oryzae* and to increase their density in a concentration-dependent manner. The salt-controlled submerged cultivation leverages the induction of osmolarity-driven effects to control fungal pellet size. Wucherpfennig et al. (2011) saw a clearly inverse correlation between increasing NaCl concentrations and pellet size and a direct correlation of increased enzyme productivity by the biomass of *Aspergillus* sp. that was cultivated in this study. Dobson and O’Shea (2008) explored to a deeper level the effects of different cations (Ca^2+^ and Mg^2+^) in diversifying the hydrophobicity of the pelletised morphology of filamentous microorganisms, where Ca^2+^ promoted a hydrophobic surface and consequential increased pelletisation, while Mg^2+^ decreased it, therefore finding that both antagonistic factors directed the pelletisation into divergent directions. Junker et al. (2004) and Zhang and Zhang (2015) collated these factors in comprehensive reviews that suggested how interplaying factors could be used to engineer the filamentous morphology and how that is intimately linked to increased biomass productivity.

## Hydrodynamics challenges for the submerged cultivation of filamentous fungi in airlift bioreactors

The two-phase and three-phase modelling (Garcia-Calvo et al. 1999; Han et al. 2017; Zhang et al 2021) that describe the hydrodynamics in the ALR is dramatically altered by the dynamic behaviour and evolution of the mycelial aggregates (pellets). This is especially the case during (semi)continuous cultivation strategies (Jin et al. 2001; Cerrone et al. 2024) and prolonged batch cultivations (Souza Filho et al. 2017; Nair et al. 2016; Furlan et al. 2024). Fragmented mycelia that have a controlled aggregation behaviour could increase both the fungal biomass density and the volumetric oxygen transfer; in addition, this factor can also promote a more efficient airlift and fluidification motions in the ALR. This is usually mirrored by an increased broth viscosity; Kilonzo and Margaritis (2004) highlighted that small bubbles (0.1 mm) have a longer contact time and bigger interfacial area and they experience a reduced superficial gas velocity in viscous non-Newtonian fluids; this feature could partially counteract the reduced oxygen availability due to the increase of pellet density and pellet volumes. Mechanical disruption is excluded by design in ALR; therefore, any aggregation behaviour of the fungal biomass is just controlled by physico-chemical phenomena of adsorption and hydrophobic interactions (other than genetic control). Retaining the pneumatic motion in the airlift can be achieved by increased superficial gas velocities (Jin et al. 1999; Botello-Alvarez et al. 2006); the efficient airlift motion of smaller mycelia fragments could also be obtained with the media alterations previously described. To the best of our knowledge, there are no studies that directly altered the cultivation media for the control of the pellet size and morphology, and subsequent scale-up in ALR. The most effective option to increase fungal biomass density by decreasing pellet sizes seems to be the introduction of a design modification into the airlift bioreactors. This design modification is usually the adoption of a mechanical agitation; this mechanical agitation can be tailored in frequency and speed, especially in cases of extremely shear stress-sensitive filamentous microorganisms. This aspect is explored in the next section.

## Design modifications of airlift bioreactors for the cultivation of filamentous fungi

**Fig. 6:**
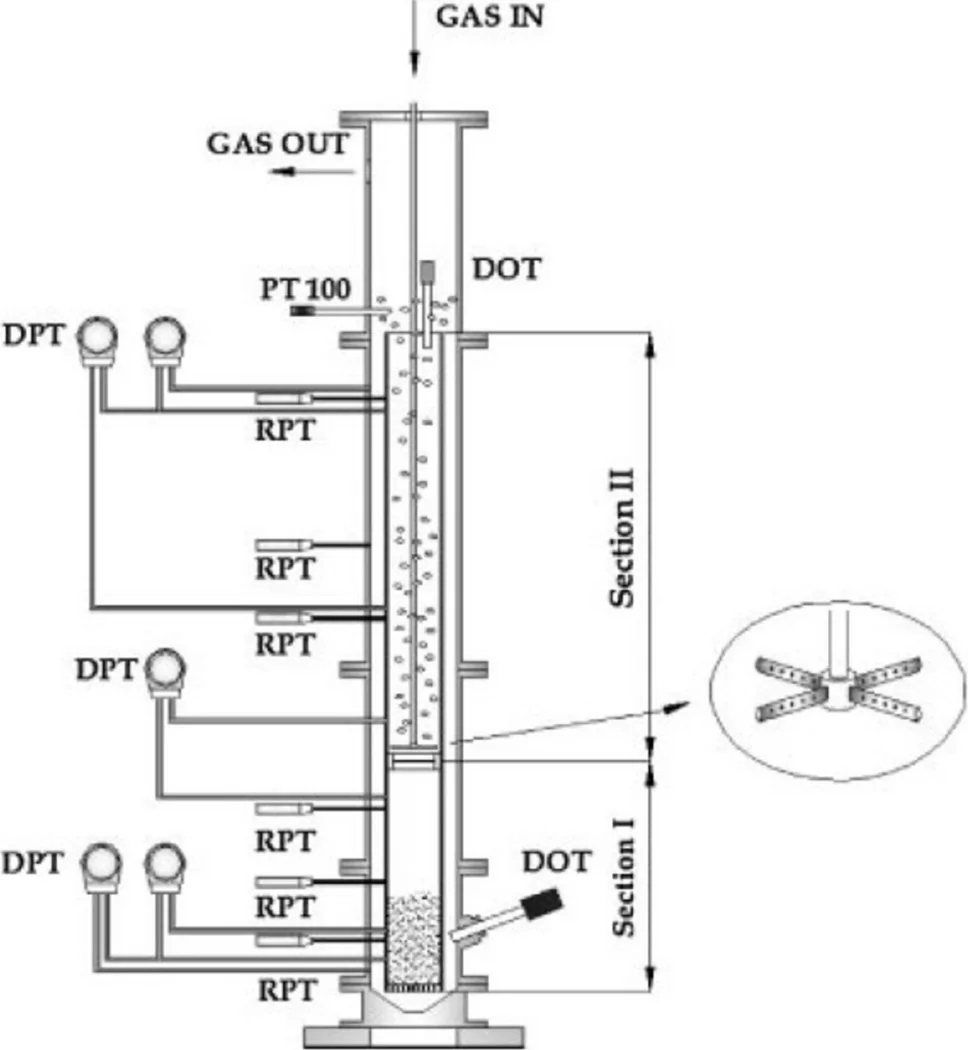
Modified airlift bioreactor with an internal sparger in the draft tube. DPT, differential pressure transducers; RPT, relative pressure transducers; DOT, dissolved oxygen probe. (inset = gas sparger design). Picture adapted from Olivieri et al. (2010)

Two major commercially produced biomass of edible microorganisms (Quorn™ and Pruteen™) (Trinci 1992) (Westlake 1986) have been produced at scale using ALR, and the bioreactor sizes were 150,000 and 1,500,000 L for a production of 14,000 tonne/annum and 50,000 tonne/annum, respectively. Due to the drawback of mycelia clumping, many researchers further explored ALR modifications to enhance their performance. Ahamed and Vermette (2010) saw a clear reduction in hyphal entanglement, volume, and length when they introduced a 400-RPM stirring shaft in a 35-L ALR; but they also saw a decreased biomass concentration (20%) and a reduction in enzymatic productivity (25%), with a concomitant 50% increase in medium viscosity caused by the mechanical agitation. Park et al. (1994) successfully focused on the fermentation strategy of cell recycling for the ALR (semi)continuous cultivation of fungal biomass; even if their product volumetric productivities were high (0.47 g/L/h), they noticed an impaired airlift motion in the long term (after 45 days of cultivation) due to adhesion of agglomerated mycelia clumps. An ingenious solution of a pulsating airflow in ARL was attempted by Moreira et al. (1996); these researchers found out that extremely low frequency pulsating airflow (0.0625 Hz) produced pellets of *Phanerochaete chrysosporium* with a reduced diameter (2 mm vs 3 cm) and more than doubled the duration of the fermentation (14 vs 34 days), compared to a non-pulsating airflow. Olivieri et al. (2010) also explored a modified airflow dynamic, and they located the sparger at 1/3 height of the draft tube (Fig. 6) allowing an intentional separation between the solid/liquid phase (below) from the gas/liquid phase (above); this modification induced a liquid-only driven recirculation for the suspended particles and a gas-driven recirculation for the media, and the advantage of this configuration is due to the reduction of oxygen gradients that could be caused by the differential absorption of oxygen by the suspended solids; the researchers claim that with this configuration, the media aeration is more homogenous and the fluidisation is more effective; this modification can be especially useful for slow-growing microorganisms.

**Fig. 7:**
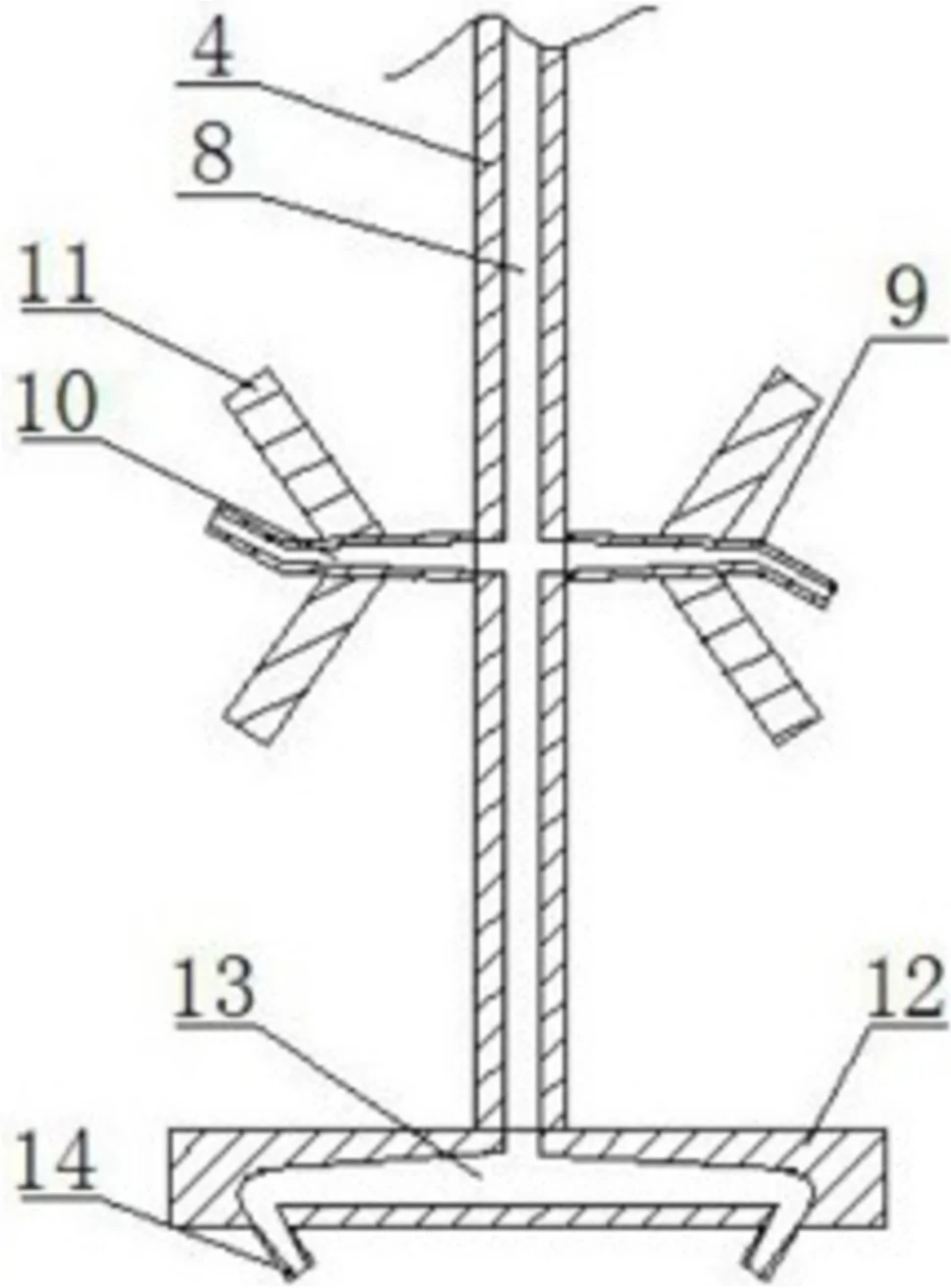
Air supply rotating shaft system for ALR for the cultivation of filamentous fungi *(Armillaria mellea)* where the individual components described as follows: (4) rotating shaft, (8) air supply inlet, (9) downward conical jet-head, (10) upward conical jet-head, (11) additional rotating blades, (12) lower jet-head, (13) bottom portion of the sparger, (14) bottom jet nozzle. Picture adapted from Peng (2022)

ILALR and ELALR are the two major configurations for ALR; both options have been proven successful for the cultivation of filamentous fungi as previously mentioned. The introduction of mechanical agitation in ALR for the cultivation of filamentous fungi took multiple different variations; the patent (CN216919223U), merged the use of a jet air supply through the stirring shaft, designing a rotating air supply system (Fig. 7). The inventors claim this ALR modification allows high cell density of *Armillaria mellea* strain, specifically by increasing the surface area of the air bubbles and by increasing the gas velocity.

**Fig. 8:**
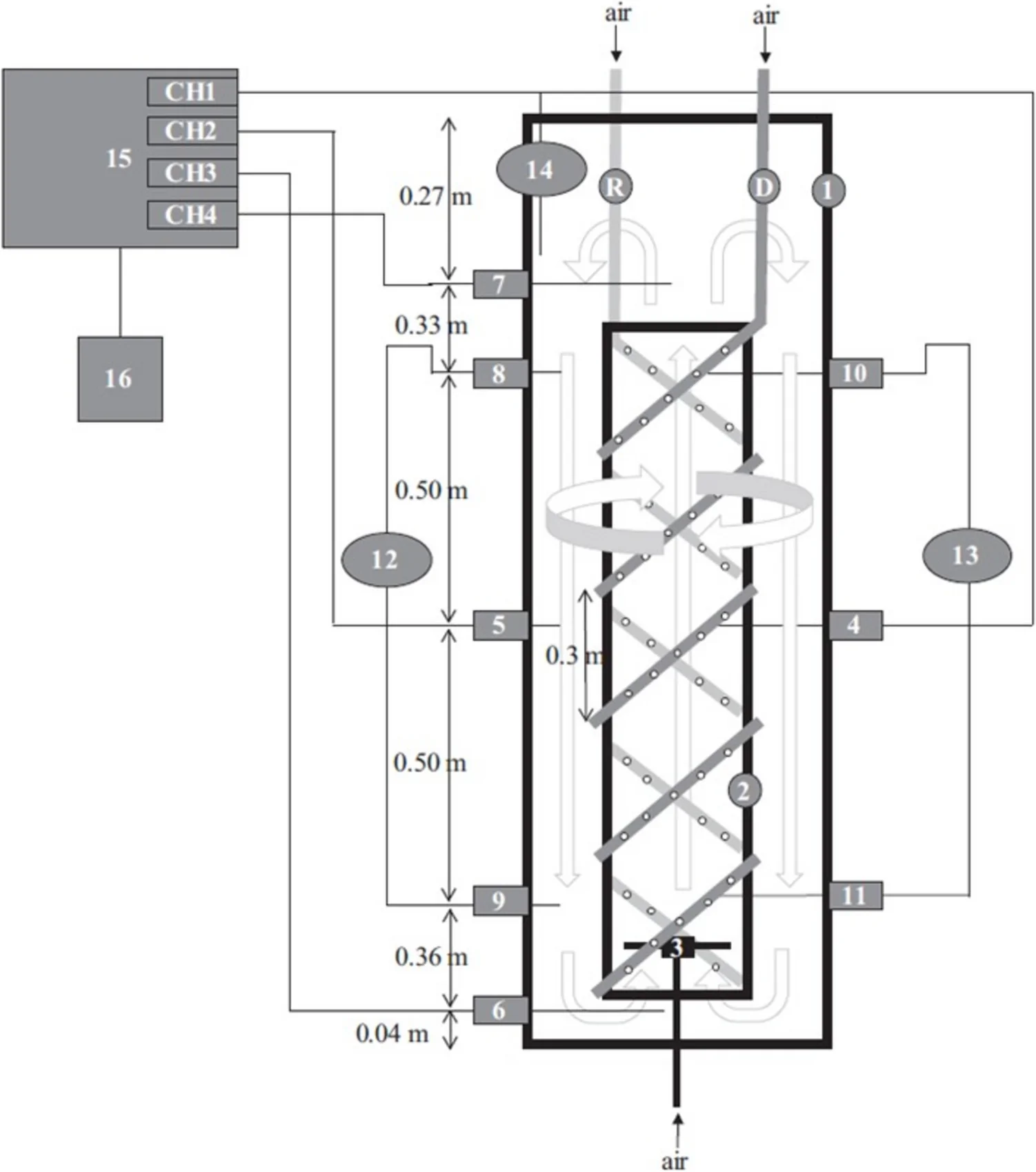
(1) Draft tube, (2) gas sparger, (3) DO-electrodes: middle riser, (4) middle downcomer, (5) base, (6) gas separator, (7) pressure taps/perforated tubes in downcomer, (8) (9) pressure taps/perforated tubes in riser, (10) (11) inverse U-tube manometers, (12) (13) temperature probe, (14) Biostat Q DCU, (15) PC/recorder, (16) riser helix/HFPgas sparger (R), downcomer helix/HFP-gas sparger (D). Picture adapted from Räsänen et al. (2016)

Räsänen et al. (2016) introduced a helical shaped flow promoter (HFP) inside an ALR, as a mechanism to promote ALR performance and fluidisation of suspended pelletised particles. The HFP was dual and located outside the draft tube (in the downcomer) and inside the draft tube (in the riser); both HFP were connected to a different air supply port (Fig. 8).

**Fig. 9:**
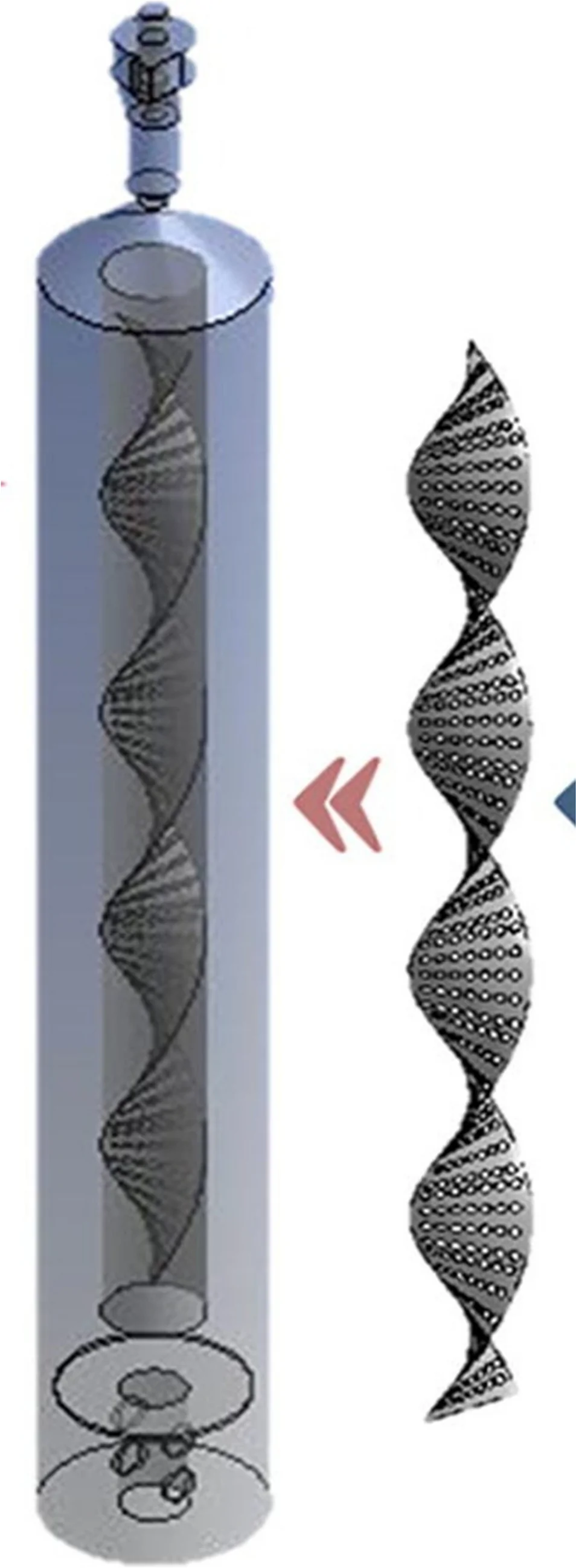
Helical agitator in draft tube of an airlift bioreactor. Picture adapted from Sayyar et al. (2023)

Their finding highlights that the HFP in the downcomer is especially effective in increasing the K_L_a of the system (a threefold increase when compared to a regular ALR); this has been attributed to a higher gas hold-up and an increased residence time; in addition, the power/volume was almost sixfold better than compared to a STR; it is worth highlighting that these researchers did not test the modified ALR with filamentous fungi in an actual cultivation scenario. The inventors that patented the technology (CN114195252A, Wu et al. 2022) also introduced a helical modification in ALR, but in this case, the helical morphology was the shape of the agitator, located in the draft tube (Fig. 9). This system was used for the cultivation of *Trichosporon*. A similar solution was also proposed by US2018/0119083A1, Zheng et al. (2018) where a perforated helical stirrer was located either in the draft tube or surrounding the draft tube (in the downcomer space). In both cases, this mechanical agitation accelerates the gas velocity of the supplied air and progressively decreases the bubble sizes, preventing coalescence phenomena.

**Fig. 10:**
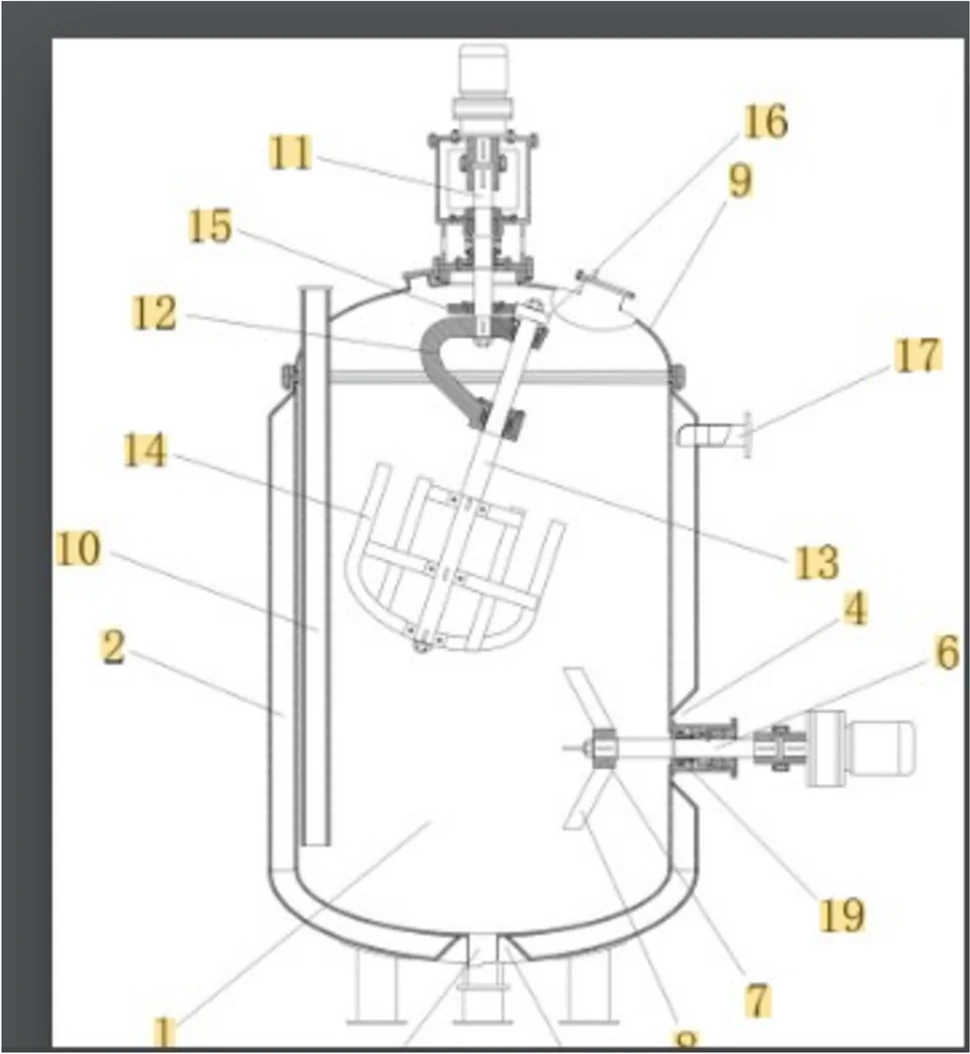
Airlift bioreactor provided with a tilted low-intensity anchor-shaped agitator (13). Picture adapted from Zhang et al. (2020)

Researchers that patented the ALR design (CN211947007U) also considered an ALR modification related to mechanical agitation; their anchor-shaped solution was composed of a dual set of stirrers; the first central shaft holds a 20° inclined and partially suspended secondary shaft and both sit inside an ALR (Fig. 10). This solution takes advantage of the sweeping effect of the tilted shaft and greatly decreases the chances of fungal biomass adhesion, preventing clumping. Chisti and Jauregui-Haza (2002) also introduced a mechanical agitator, composed of Prochem® hydrofoil impeller blades; according to these researchers, this feature only increased the mixing of the medium but did not improve the volumetric oxygen transfer when with high mycelia density. Tekić et al. (2014) introduced regularly spaced blades along a central shaft in the draft tube (riser) of an ALR; they found out that this feature specifically increases the gas hold-up in the riser, but it has a detrimental effect on the gas hold-up inside the downcomer.

**Fig. 11:**
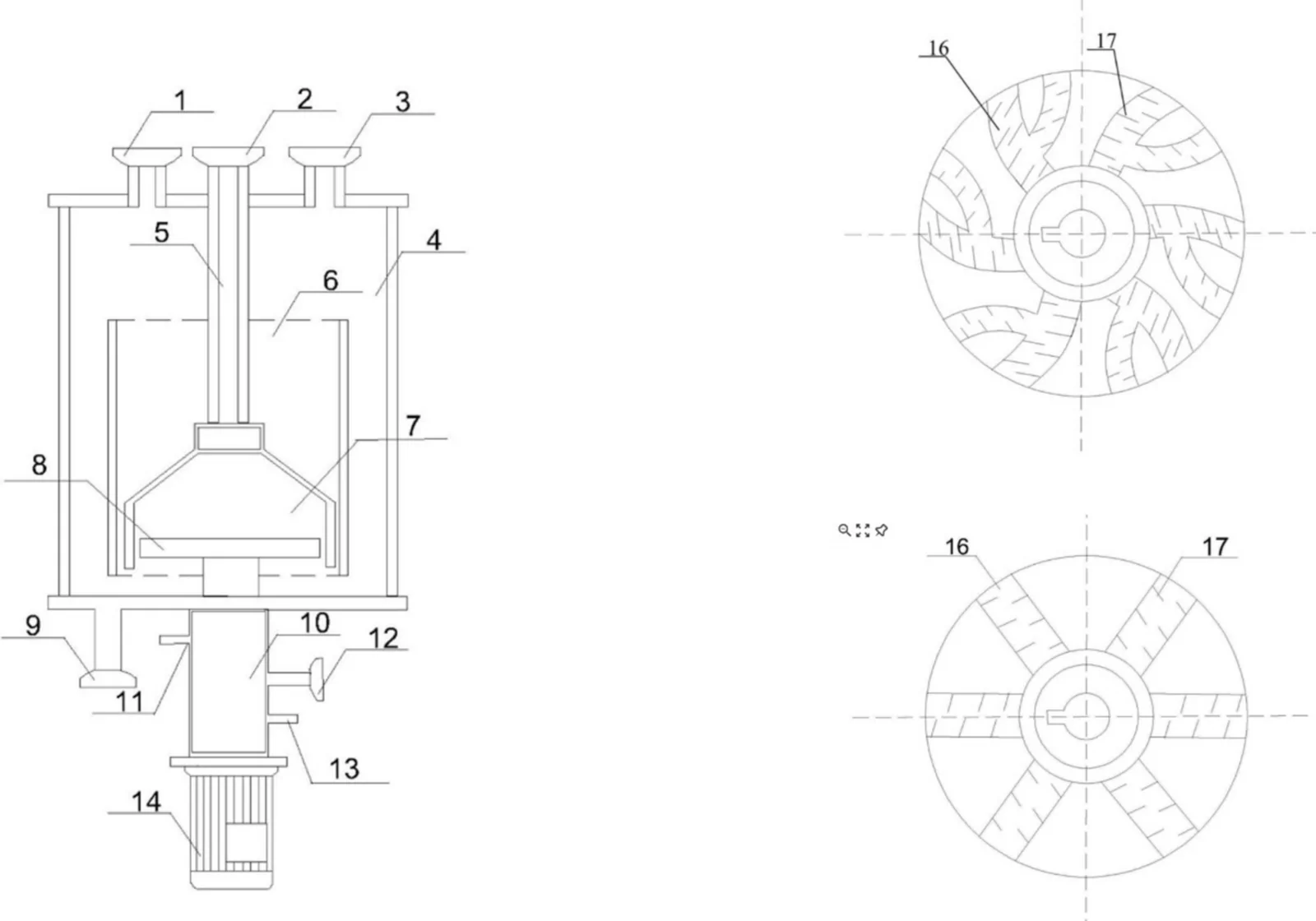
(1) liquid inlet, (2) upper air inlet, (3) air outlet, (4) reaction tank, (5) air guide pipe, (6) guide tube, (7) air guide hood, (8) self-priming impeller, (9) drain, (10) seal, (11) cooling water inlet, (12) lower air inlet, (13) cooling water outlet, (14) generator, (15) screen, (16) gas–liquid flow channel, (17) baffles. Picture adapted from Li et al. (2022)

Other researchers specifically tailored their patent (CN1148438C) for the cultivation of filamentous fungi in ALR; the main novel modification they introduced was a set of spray nozzles to perform a wall cleaning mechanism (by pressurised sterile water) and reduce mycelia adhesion on the walls of the vessel. Some researchers, Gong et al. (2004) patented a design (CN114307935A), which uses a novel air supplier for an ALR. They designed a mixer system that conveys the air supply towards a collecting submerged hood, using an externally located multi-channel centrifugal impeller. This self-suction device greatly reduces the size of the sparged bubbles, preventing their coalescence and maximising their surface area (Fig. 11). In addition to that, they also envisaged two types of baffled spargers to further increase the surface area of the supplied air (Fig. 11). Liu et al. (2010) and Fadavi and Chisti (2005) enhanced the gas supply by using a forced circulation in ILALR and they both saw a 30% improvement in K_L_a through the creation of a bigger interfacial area of the gas supply.

**Fig. 12:**
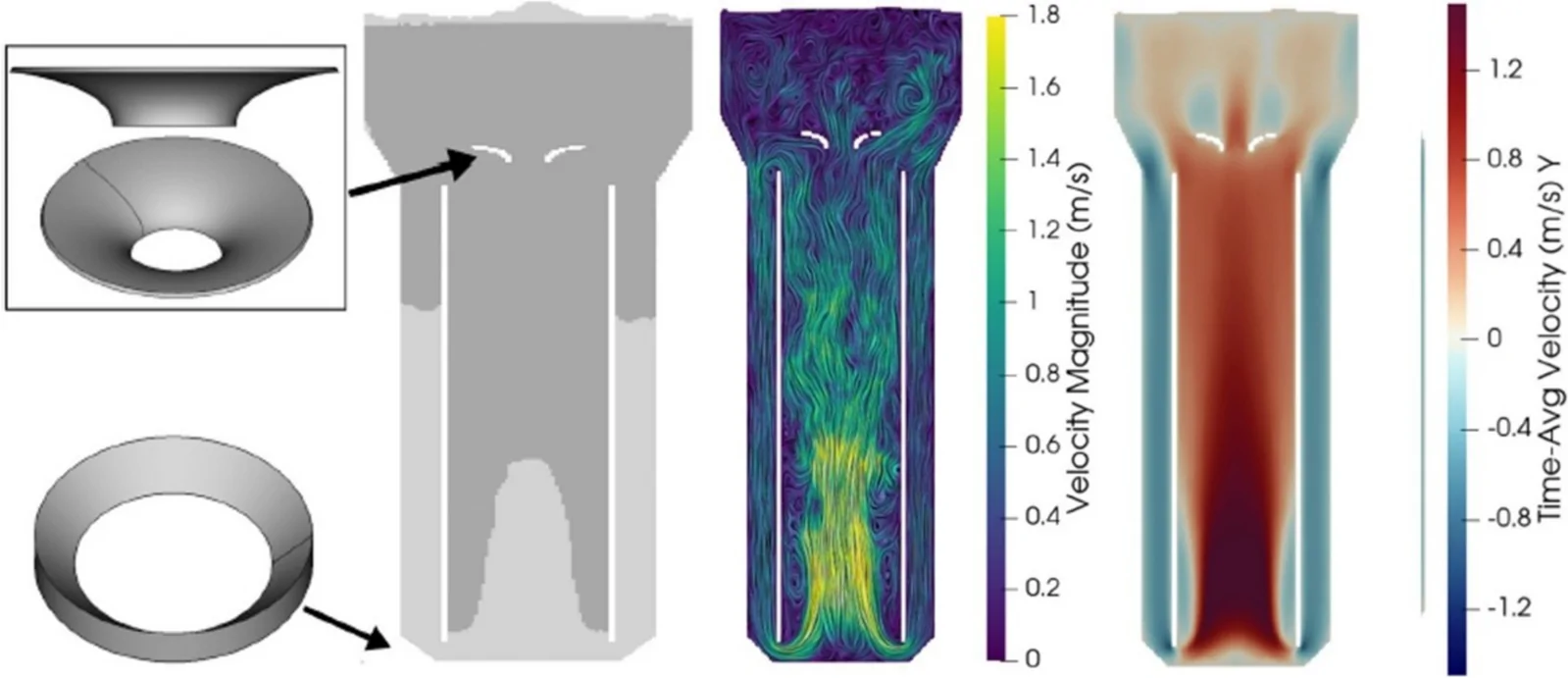
Computational fluid dynamics of a CR-ILR modification with internals. (From left to right): visualisation of the design of the internals added to a CR-ILR, line integral convolution of the velocity magnitude, and vertical velocity profile of the airflow in a CR-ILR. Picture adapted from Bokelmann et al. (2025)

Bokelmann et al. (2025) performed a c-mparative in silico study by computational fluid dynamics, where they considered a bubble column reactor (BCR), an annulus rising internal loop airlift (AR-ILR), a centre rising internal loop airlift (CR-ILR), and an ELALR (being the latter the most used in industrial settings). All of these systems were provided with conical internals to enhance their K_L_a. The CR-ILR showed a superior oxygen mass transfer when provided with internals in the top clearance area (Fig. 12). Despite this study highlighting the importance of performing a priori in silico simulations also for ALR modifications, these findings were not further tested in a wet-lab scenario for the cultivation of filamentous fungi.

Additional modifications focus on the introduction of selective membranes within the ALR; the objective is dual: retain/recirculate the filamentous fungi biomass and maximise catabolite exchange. CN109735452A, Chen et al. (2019) introduced a specific membrane provided with tubing to facilitate microbubble delivery and increase the interfacial area of the gas supply. CN212476267U, Huang et al. (2021) introduced a further modification in a membrane-based ALR by using electrochemically active membranes; this device offers a dual purpose: it enhances the flocculation of the biomass and promotes a more efficient electron transfer for biological respiration, even if this last patent was not directly tailored for filamentous fungi cultivation. A final ALR modification explored by some researchers was the introduction of pulsed ultrasound technology (CN116478813A, Ma et al. 2023) using hollow baffled transducers for the enhancement of the microbial growth and for the further increase of nutrients and oxygen uptake. Even if this solution is not directly described as useful for the enhancement of the mycelia density and/or volumetric productivity in ALR, there are studies that use ultrasound technology for the separation of filamentous fungi (Crognale et al. 2002); therefore, there is a possibility to also explore this modification in an ALR setting.

All the above-mentioned studies that worked in the research area of cultivation of filamentous fungi in ALR demonstrated that despite common adhesion phenomena (induced by clumped aggregation during prolonged fermentations), the modification with a mechanical agitation does not necessarily improve the biomass volumetric productivities or the efficiency of the system.

## Helpful lessons from the cultivation of different filamentous microorganisms in airlift bioreactors

Studies about the use of ALR for the cultivation of filamentous fungi usually focus on a two-prong approach: (1) exploring the enhancement of the ALR performance through a mixture of physical experiments and computational modelling; these studies especially focus on increasing the K_L_a, and the gas hold-up of the ALR; (2) exploring the metabolic adaptations of filamentous fungi within a low-intensity agitation promoted by pneumatic motion and trying to infer performance parameters (yields and volumetric productivities) that are linked to the morphological shape of the pelletised aggregates in submerged cultivation (especially linking it with oxygen availability). Very little scientific literature is available regarding studies that can integrate these two approaches in a comprehensive manner. There is a lack of studies that have a deep understanding of hydrodynamics modelling of a three-phase system and especially regarding prolonged continuous fermentations that could demonstrate scaling potential. Most studies either describe batch cultivation of submerged fungal biomass, even at high cell density but without a proper understanding of the best design and hydrodynamics of an ALR or, conversely, they only demonstrate the engineering aspects of ALR optimisation in simulation scenarios. It is likely that the complexities of both approaches demand a high number of scientific resources to perform such comprehensive studies.

## Conclusions

To sum up, these are the major conclusions that can be derived from ALR cultivation of filamentous fungi:

1. A careful understanding of the overall parameters involved in the ALR operation and especially their dynamic interplay in prolonged cultivation is beneficial for a comprehensive modelling (where computational fluid dynamics (CFD) and process analytical technologies (PAT) could be extremely helpful for a high-resolution modelling, but they both require high computational power).
2. Batch cultivation of filamentous fungi in ALR is particularly successful for enzymatic production, low-medium cell density, medium/high cell density with low volumetric productivities, and the biodegradation of recalcitrant compounds.
3. A somehow controllable degree of pelletisation of the filamentous fungi can enhance biomass density, maximise yields, and enhance biomass volumetric productivities, especially if the homogenous pellet size can be controlled (by pH, genetic control, media composition or ALR design modifications).
4. Mechanical disruption of pelletised fungal biomass not necessarily increases the volumetric productivities, but it helps in increasing nutrients mixing and increase of interfacial area of the supplied gas. Specific power/volume considerations need to be performed for scaling purposes if a mechanical agitation modification is introduced in ALR.
5. ALR cultivation of filamentous fungi has been repeatedly demonstrated at scale (MTonn scale), and it is justified by the enhanced volumetric oxygen transfer but is limited to very few model organisms (*Aspergillus, Fusarium*), and to date, only one study has focused on prolonged semi-continuous cultivation of *Basidiomycota* in ALR, specifically for biomass production.
6. Specific considerations and bioreactor designs are needed if the filamentous fungi are the targeted product or if they are just the biocatalysts of the bioprocess. ALR is possible for both.
